# Cytogenomics Unveil Possible Transposable Elements Driving Rearrangements in Chromosomes 2 and 4 of *Solea senegalensis*

**DOI:** 10.3390/ijms22041614

**Published:** 2021-02-05

**Authors:** María Esther Rodríguez, Ismael Cross, Alberto Arias-Pérez, Silvia Portela-Bens, Manuel Alejandro Merlo, Thomas Liehr, Laureana Rebordinos

**Affiliations:** 1 Área de Genética, Facultad de Ciencias del Mar y Ambientales, Instituto Universitario de Investigación Marina (INMAR), Universidad de Cádiz, 11510 Cádiz, Spain; mariaesther.rodriguez@uca.es (M.E.R.); ismael.cross@uca.es (I.C.); alberto.arias@uca.es (A.A.-P.); silvia.portela@uca.es (S.P.-B.); alejandro.merlo@uca.es (M.A.M.); 2 Institute of Human Genetics, University Clinic Jena, 07747 Jena, Germany; thomas.liehr@med.uni-jena.de

**Keywords:** *Solea senegalensis*, cytogenomics, chromosome evolution, comparative genomic, repetitive sequences, robertsonian fusions, pleuronectiformes

## Abstract

Cytogenomics, the integration of cytogenetic and genomic data, has been used here to reconstruct the evolution of chromosomes 2 and 4 of *Solea senegalensis*. *S. senegalensis* is a flat fish with a karyotype comprising 2n = 42 chromosomes: 6 metacentric + 4 submetacentric + 8 subtelocentric + 24 telocentric. The Fluorescence *in situ* Hybridization with Bacterial Artificial Chromosomes (FISH-BAC) technique was applied to locate BACs in these chromosomes (11 and 10 BACs in chromosomes 2 and 4, respectively) and to generate integrated maps. Synteny analysis, taking eight reference fish species (*Cynoglossus semilaevis*, *Scophthalmus maximus*, *Sparus aurata*, *Gasterosteus aculeatus*, *Xiphophorus maculatus*, *Oryzias latipes*, *Danio rerio,* and *Lepisosteus oculatus*) for comparison, showed that the BACs of these two chromosomes of *S. senegalensis* were mainly distributed in two principal chromosomes in the reference species. Transposable Elements (TE) analysis showed significant differences between the two chromosomes, in terms of number of loci per Mb and coverage, and the class of TE (I or II) present. Analysis of TE divergence in chromosomes 2 and 4 compared to their syntenic regions in four reference fish species (*C. semilaevis*, *S. maximus*, *O. latipes,* and *D. rerio*) revealed differences in their age of activity compared with those species but less notable differences between the two chromosomes. Differences were also observed in peaks of divergence and coverage of TE families for all reference species even in those close to *S. senegalensis,* like *S. maximus* and *C. semilaevis*. Considered together, chromosomes 2 and 4 have evolved by Robertsonian fusions, pericentric inversions, and other chromosomal rearrangements mediated by TEs.

## 1. Introduction

Cytogenomics is a methodology in which the cytogenetic and genomic data obtained are integrated. This approach emerged to advance studies of the relationship between chromosomes and diseases in humans, but it has been extended to other species due to its potential value for studies of evolution [1]. The wide variability of karyotype observed among species in cytogenetic studies has prompted researchers to investigate the molecular mechanisms that underlie chromosome structure and function. Next Generation Sequencing (NGS) techniques have allowed us to characterize the genome of many organisms and to perform sequence-based comparisons between them. The integration of sequencing and mapping data across the genome is helping us to visualize past rearrangement events and to assess synteny among species [2]. The availability of more data from a greater number of species should help to clarify the apparent relationship between intra- and interspecific variation and its relationship to environmental conditions.

Comparative genomics of orthologous sequences located in the karyotype has opened new ways to detect karyotype differences because it allows comparisons to be made between species, genera, and even families, providing a more detailed overview of the evolutionary changes in the karyotype. It also allows us to overcome serious limitations of cytological studies such as genetic disruption of meiotic pairing and indistinguishable chromosomes [3,4].

A large part of the eukaryotic genome is composed of repetitive DNA including satellite DNA, and transposable elements (TE). The majority of these repetitive non-coding sequences are usually located in heterochromatic regions such as centromeres and telomeres, and in other specific regions of the genome. They are organized into blocks of DNA, scattered interspersed repetitions, that undergo periodic reorganization mostly due to mobile elements that jump from one location to another, often leaving a copy of themselves in the original location. Hence the evolutionary dynamics of TEs are important, not only for genome size but, because the regrouping of mobile elements can originate differences in the number and structure of the chromosomes in some species [5].

According to the transposition mechanism there are two types of transposons: those with and those without an intermediate RNA. That difference divides TEs into class I and class II. Class I TEs are the retrotransposons that produce an RNA intermediate by reverse transcription that is moved through the genome by a copy-paste mechanism. This class is subdivided into Long Terminal Repeat (LTR) and non-LTR retrotransposons. Class II transposons or DNA transposons can utilize three transposition mechanisms: first, “cut-and-paste” transposons; second, inverted terminal repeat sequence (ITRs) transposons (e.g., hATs and Helitrons); and third, self-synthesizing DNA transposons (e.g., Mavericks) [6]. Among vertebrates, teleosts have the highest number of TE superfamilies, and their abundance seems to be determinant of the size of the genomes of this group, in spite of the smaller size of genomes in fishes, indicating great variability between the species [7]. Among actinopterygians, teleost fishes present the greatest diversity, with more than 25 TE superfamilies described in some species (Gypsy, BEL/Pao, ERV, DIRS, Penelope, Rex6/Dong, R2, LINE1, RTE, LINE2, Rex1/Babar, Jockey, Helitron, Maverick, Zisupton, Tc-Mariner, hAT, Harbinger, PiggyBac, and EnSpm) [8,9].

Fishes show the highest chromosome number variation among vertebrates due to an extensive evolutionary radiation. Among them, the teleost group, which emerged 225 mya, is extremely diverse in terms of morphology, behavior, and adaptation [10]. This heterogeneity could be related to a whole-genome duplication, in addition to the two duplications that occurred during the origin of vertebrates, and that took place in this group before it diversified [11]. Several different lines of evidence, such as the number of chromosomal rearrangements, functionalization of duplicated genes, rate of protein evolution, and conservation of non-coding elements, show a higher rate of evolution in teleosts than in other vertebrates. This group presents small chromosomes and an ancestral karyotype of 48 acrocentric chromosomes [12,13].

The Pleuronectiformes order, in particular, has undergone intense chromosome evolution. The phylogeny of this order has been disputed, with some studies supporting a monophyletic origin [14,15] and others a poly- or paraphyletic one [16,17,18]. Recently, the analysis of more than 1000 UCE (Ultraconserved DNA Elements) loci provided strong molecular support for the monophyletic origin of flatfishes and for the single origin of cranial asymmetry [19]. Pleuronectiformes present flattened bodies as the result of a metamorphosis process that converts a symmetric larva into an asymmetric juvenile. They live close to the sea bottom and show extreme adaptation to this environment. This order is usually divided into seven families, of which four are of commercial importance: Pleuronectidae, the right eye flounders; Scophthalmidae, the left eye topknots; and Soleidae and Cynoglossidae, the soles.

The genome size of flatfishes is among the smallest of all fishes; a size of 612.3 Mbp has been reported for *S. senegalensis* [7] and an even smaller size in *Cynoglossus semileavis* with 470 Mbp [20,21,22]. *Danio rerio* with 1371 Mpb is more than double and nearly triple the size of the latter two species, respectively [23,24]. Their chromosomes are very small [25]: the chromosome diploid number ranges from 2n = 28, observed in the Paralichthyidae *Citarichthys spilopterus*, to 2n = 48, found in most of the Pleuronectidae species [21]. This variability has been explained by the occurrence of Robertsonian fusions and chromosome inversions during the course of the evolution of Pleuronectiformes [21,26].

The Senegalese sole (*Solea senegalensis* (Kaup, 1858)) is a flatfish, with an oval and asymmetric body, belonging to the Pleuronectiformes order. The species is widely distributed in the Atlantic, from the Gulf of Biscay to the Northwest coast of Africa, and in Mediterranean waters, from the Strait of Gibraltar to Tunisia; this species has good potential for marine aquaculture given the high demand and profitable price. However, there are several issues that hamper its production: (1) high larval mortality, (2) sub-optimal larval weaning strategies, and (3) disease control. In recent years, considerable efforts have been devoted towards understanding genetic and genomics aspects of this species. The karyotype of this species comprises 2n = 42 chromosomes: 6 metacentric (M) + 4 submetacentric (SM) + 8 subtelocentric (ST) + 24 telocentric (T), and its Fundamental Number (FN) is 60 [27]. Its chromosomes are very small with sizes ranging from 0.5 to 1 µm [28]. The largest metacentric chromosome, chromosome 1, has been proposed as a sex proto-chromosome originated through a Robertsonian fusion [3]. The rest of the chromosomes have BAC-based markers that enable them to be distinguished [9] and its characterization can contribute important clues, taken in consideration that it is a non-model species with limited genomic resources available, and also to contribute to the karyotype evolution of Pleuronectiformes. Hence, the object of the present paper is to study the evolution of chromosomes 2 and 4 of *S. senegalensis* (classified as metacentric and submetacentric, respectively) by comparing them with other available species and analyzing their repetitive elements.

## 2. Results

### 2.1. Description of the Metacentric and Submetacentric Chromosomes 2 and 4

**Figure 1:**
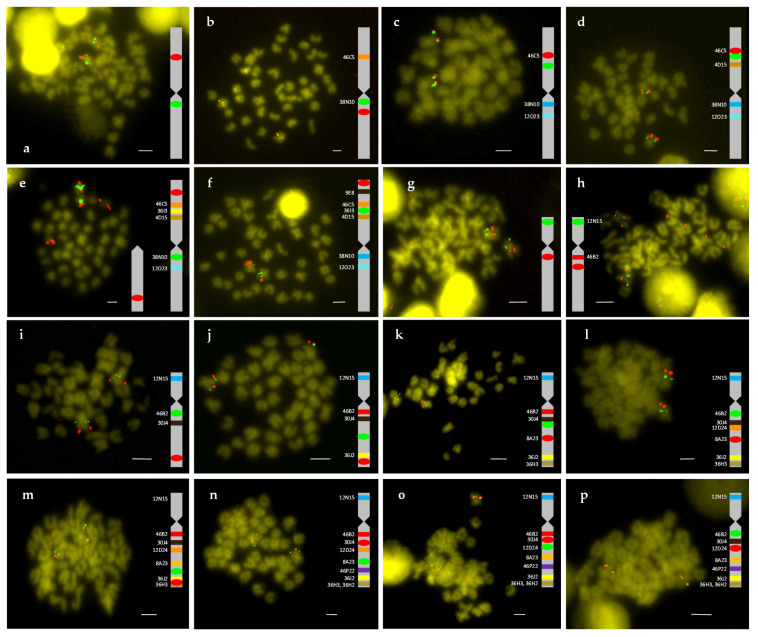
Double FISH-BAC of chromosome 2 (**a**–**f**) and chromosome 4 (**g**–**p**). (**a**) 38N10 (green)/46C5 (red), (**b**) 38N10 (green)/21O23 (red), (**c**) 4D15 (green)/46C5 (red), (**d**) 36I3 (green)/46C5 (red), (**e**) 38N10 (green)/9E8 (red), (**f**) 36I3 (green)/52G10 (red), (**g**) 12N15 (green)/46B2 (red), (**h**) 12N15 (green)/30J4 (red), (**i**) 46B2 (green)/36J2 (red), (**j**) 8A23 (green)/36H3 (red), (**k**) 12D24 (green)/8A23 (red), (**l**) 46B2 (green)/8A23 (red), (**m**) 46P22 (green)/36H2 (red), (**n**) 8A23 (green)/30J4 (red), (**o**) 12D24 (green)/30J4 (red), and (**p**) 46B2 (green)/12D24 (red). Bar = 2 μm (referred to large metacentric chromosome 1).

**Figure 2:**
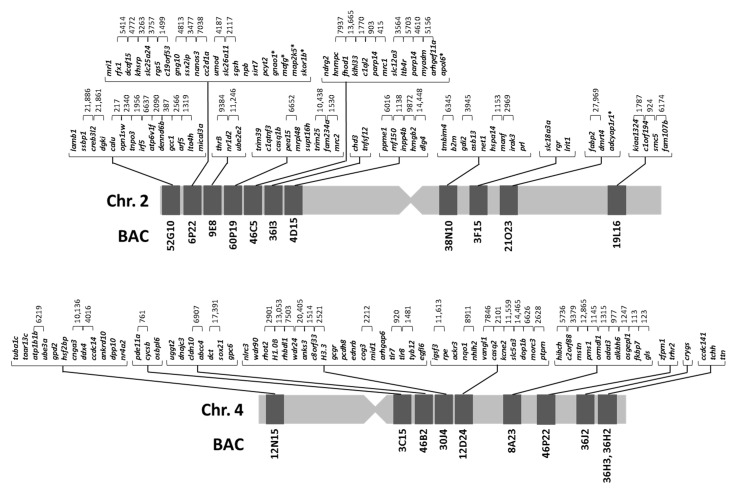
Integrated genetic maps of metacentric chromosome 2 and submetacentric chromosome 4 of *Solea senegalensis*.

A total of 21 BACs with 154 genes were annotated (Table 1). The relative position of these BACs in each chromosome was obtained by double FISH-BAC (Figure 1). The integrated maps were generated from analysis of cytogenetic, BAC sequencing and micro-synteny data (Figure 2). These maps were used to study synteny as described below. For metacentric chromosome 2, 7 out of 11 BACs (52G10, 6P22, 9E8, 60P19, 46C5, 36I3, and 4D15) were positioned in arm 1 (in a telomere to centromere direction, 66 genes annotated). The other 4 BACs (38N10, 3F15, 21O23 and 19L16) were placed in arm 2 (in a centromere to telomere direction, 19 genes annotated). For the submetacentric chromosome 4, only 1 out of 10 BACs was positioned in the “p” arm (BAC12N15, 12 genes annotated), and the rest (3C15, 46B2, 30J4, 12D24, 8A23, 46P22, 36J2, 36H3, and 36H2) were situated in the “q” arm (in a centromere to telomere direction, 57 genes annotated).

### 2.2. Synteny Analysis for Metacentric Chromosome 2

**Figure 3:**
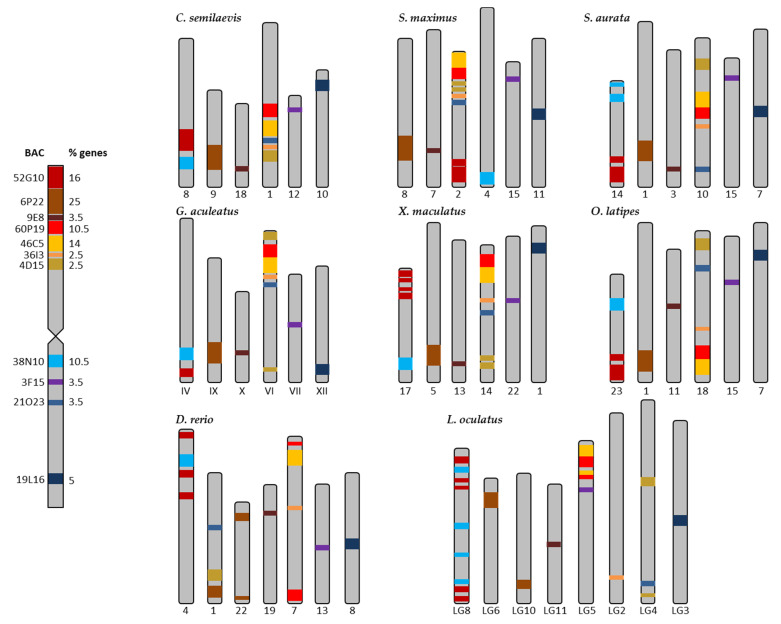
Distribution of the BACs of the metacentric chromosome 2 of *Solea senegalensis* in the chromosomes of the eight reference fish species: *Cynoglossus semilaevis*, *Scophthalmus maximus*, *Sparus aurata*, *Gasterosteus aculeatus*, *Xiphophorus maculatus*, *Oryzias latipes*, *Danio rerio,* and *Lepisosteus oculatus*. The colors represent the different BACs. % of genes is referred to the number of genes in each BAC, respective to the total of the genes in chromosome 2 (85 genes).

The synteny analysis for the eight species used as reference is presented in Figures S1–S8, and summarized in Figure 3. The taxonomic relationships of these species are shown in Figure S9. A high degree of conservation in different regions for the genes was observed in each BAC of *S. senegalensis*, and 7 out of 11 BACs were distributed in two chromosomes, except in *D. rerio* and *L. oculatus*. BACs 60P19, 46C5, 36I3, and 4D15 (arm 1 of *S. senegalensis*) and BAC 21O23 (arm 2) were located in the same chromosome in *C. semilaevis*, *S. maximus*, *G. aculeatus*, *X. maculatus*, and *O. latipes*, and in the same genomic region for *C. semilaevis* and *S. maximus*. BACs 52G10 and 38N10, which were positioned in different arms on chromosome 2 of *S. senegalensis*, were located in a second different chromosome in all species, except in *S. maximus* where they were located in two different chromosomes. For all species, gene regions for BACs 60P19 and 46C5 showed a high degree of conservation, except for *D. rerio* in which six out of nine genes of 60P19 were located in the chromosome far from the BAC 60P19, and only one gene of BAC 46C5 was located near BAC 60P19 (see Figure S7). The other BACs mapped to chromosome 2: 6P22, 9E8 (both situated in arm 1), 3F15, and 19L16 (in arm 2) were located in four different chromosomes, except *D. rerio* and *L. oculatus*, in which the genes of BAC 6P22 were distributed in two different chromosomes. A comparative mapping graph plot (Figure 5a) was used to distribute the nodes (BACs and syntenic fish species chromosomes) in the plane. Nodes sharing more connections (syntenic positions with other fish chromosomes) are closer to each other. Figure 5a shows two BACs (9E8 and 19L16) with single connections to chromosomes from other fishes, three BACs (3F15, 6P22, and 38N10) with a few connections and six BACs (52G10, 60P19, 46C5, 36I3, 4D15, and 21O23) with multiple connections, showing the conserved syntenic regions in other fish chromosomes (Figure 5a).

### 2.3. Synteny Analysis for Submetacentric Chromosome 4

**Figure 4:**
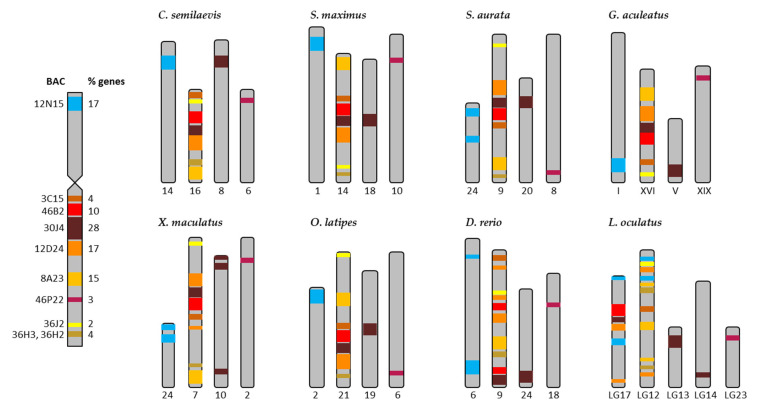
Distribution of the BACs of the submetacentric chromosome 4 of *Solea senegalensis* in the chromosomes of the eight reference fish species: *Cynoglossus semilaevis*, *Scophthalmus maximus*, *Sparus aurata*, *Gasterosteus aculeatus*, *Xiphophorus maculatus*, *Oryzias latipes*, *Danio rerio,* and *Lepisosteus oculatus*. The colors represent the different BACs in the chromosome 4. % of genes is referred to the number of genes in each BAC, in respect to the total of the genes in chromosome 4 (69 genes).

**Figure 5:**
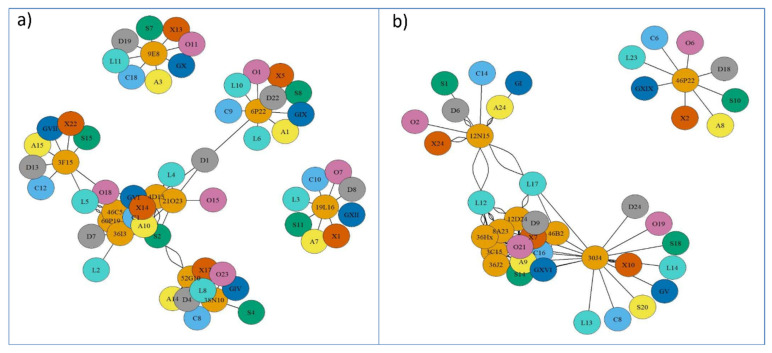
Comparative mapping graph plot of *Solea senegalensis* in (**a**) chromosome 2 and (**b**) chromosome 4. Nodes represent BACs of *S. senegalensis* (orange color) and reference fish species and their chromosomes: *Cynoglossus semilaevis* (letter C, sky blue color); *Scophthalmus maximus* (letter S, green color); *Sparus aurata* (letter A, yellow color); *Gasterosteus aculeatus* (letter G, blue color); *Xiphophorus maculatus* (letter X, vermilion color); *Oryzias latipes* (letter O, purple color); *Danio rerio* (letter D, gray color), and *Lepisosteus oculatus* (letter L, turquoise color). Edges represent presence (number) of *S. senegalensis* BAC sequences in the other fish chromosomes.

The results derived from the synteny analysis of chromosome 4 are presented in Figures S10–S17, and summarized in Figure 4. In total, 8 out of 10 BACs (3C15, 46B2, 30J4, 12D24, 8A23, 36J2, 36H3, and 36H2) mapped to the “q” arm and were located in one chromosome for all reference species. BAC 12N15, placed in the “p” arm, was located in a different chromosome in all reference species except for *L. oculatus* in which the genes of this BAC were distributed on two chromosomes. The BACs 3C15, 46B2, 30J4, 12D24, 8A23, 36J2, 36H3, and 36H2 presented in a different sequence in the chromosome in each of the species, but the region with BACs 46B2, 30J4, and 12D24 was highly conserved and the BACs followed the same sequence in all the species except for *D. rerio* and *L. oculatus*. It is noteworthy that BAC 30J4 (19 genes) showed a translocation of ten genes (*nlrc3*, *wdr90*, *rhot2*, *H1.0B*, *rhbdl1*, *wdr24*, *anks3*, *c8orf33*, *H3.3*, and *gcgr*) in all the species (see Figures S10–S17). These BACs were located on one chromosome in all species apart from *L. oculatus* in which they were positioned on two chromosomes. Thus, only nine genes from BAC 30J4 (*pcdh8*, *ednrb*, *cog3*, *mid1*, *arhgap6*, *tlr7*, *tlr8*, *tyb12*, and *egfl6*) remained in the conserved region for BACs 46B2, 30J4, and 12D24. BAC 46P22, i.e., in the “q” arm of *S. senegalensis*; in all the reference species they were positioned in a different chromosome. The mapping graph plot for chromosome 4 (Figure 5b) showed one BAC clone (46P22) with a single connection to the chromosomes of the other species; two BACs (12N15 and 30J4) had only a few connections with other fish chromosomes; and the remaining *S. senegalensis* BAC clones presented multiple connections, creating a tight cluster of nodes.

### 2.4. Distribution of Repeated Sequences

**Figure 6:**
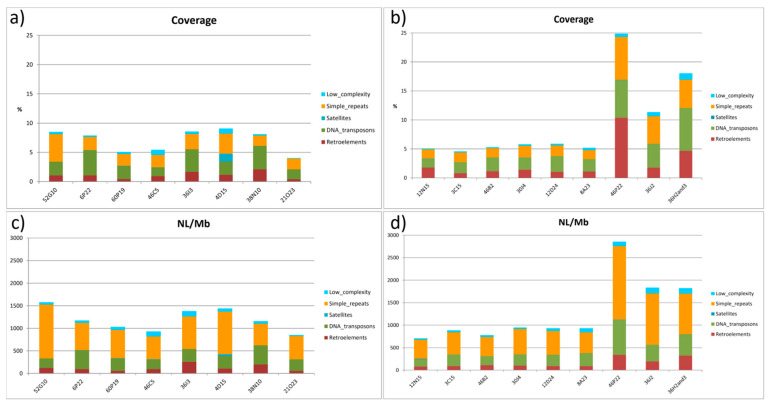
Summary of repeat types present in the BACs located in chromosomes 2 (**a**), (**c**), and 4 (**b**), (**d**) of *Solea senegalensis*. Coverage is measured as the percentage of repeat elements per BAC length. NL/Mb is the number of loci per Mb of BAC sequenced.

Chromosome 2 displayed the highest coverage values (% of repetitive elements per BAC) in the TE analysis by BAC and chromosome. Among chromosome 2 BACs, 4D15, 36I3, and 52G10 showed coverage values of 9.22, 8.54, and 8.5, respectively. BAC 52G10 presented a high percentage of simple repeats, and the presence of satellites was observed for BAC 4D15 (Figure 6a and Table S1).

The number of *loci* per Mb (NL/Mb) was proportional to coverage, but 52G10 showed higher values than the other BAC clones (1189.5 NL/Mb), mostly caused by the presence of multiple simple repeats loci (Figure 6c and Table S2). In chromosome 4, the coverage of repetitive elements was lower than in chromosome 2. However, BACs located in the subtelomeric region of the “q” arm of this chromosome (46P22, 36J2, 36H2, and 36H3) showed coverage and NL/Mb values up to five times higher than other BACs analyzed. These values correspond to both TEs and simple repeats (Figure 6b,d and Tables S3 and S4).

After grouping all BAC sequences for each chromosome, the analysis of repetitive elements was carried out. Chromosome 4 had more retroelements and with higher coverage (NL/Mb) than chromosome 2 (Table 2), mainly due to the presence of almost twice the number of LTR elements (retroviral and Gipsy/DIRs). However, DNA transposons showed higher coverage and NL/Mb in chromosome 2, mostly due to Hobo-Activator and Tc1-IS630-Pogo elements; also RTE/Bov-B LINE elements showed high values of NL/Mb and coverage in chromosome 2 (Table 2 and Figure S18).

### 2.5. Analysis of the Transposable Elements Divergence

**Figure 7:**
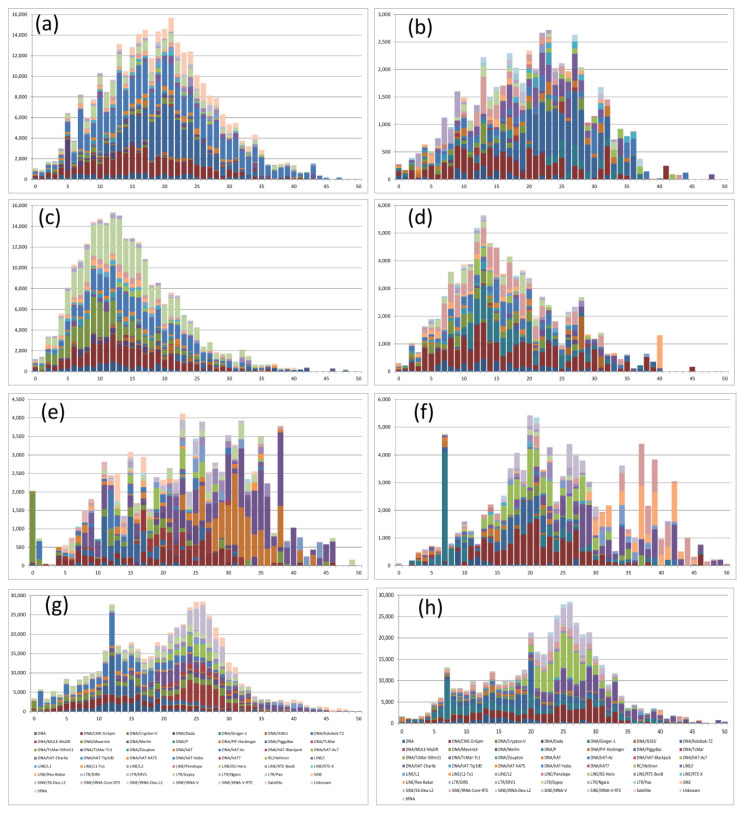
Kimura distance-based copy divergence analysis of transposable elements of chromosomes 2 and 4 of *Solea senegalensis* compared with their syntenic regions in other reference fish species. Graphs represent genome coverage (y axis) for each type of TE in the different genomes analyzed, clustered according to Kimura distances to their corresponding consensus sequence (x axis, K-value from 0 to 50). Graphs (**a**,**c**,**e**,**g**) show results from chromosome 2; and graphs (**b**,**d**,**f**,**h**) from chromosome 4. Rows are: *Cynoglossus semilaevis* (**a**,**b**), *Scophthalmus maximus* (**c**,**d**), *Oryzias latipes* (**e**,**f**) and *Danio rerio* (**g**,**h**)**.**

The most frequent Kimura’s divergence values for TEs between *S. senegalensis* chromosome 2 and syntenic regions in *C. semilaevis* ranged from 16 to 21% across all repeat classes, suggesting a relatively recent transposition burst across all major TE types. DNA/CMC/Emspm, DNA/Kolobok and DNA/hAT-Ac elements showed the highest coverage (Table 3, Figure 7a and Figure S19a).

The divergence peak of TEs in the chromosome 4 between *S. senegalensis* and *C. semilaevis* was less pronounced with higher coverage in divergences between 22 and 27% (Figure 7b and Figure S19b). The coverage over the whole divergence range was lower than that of chromosome 2, indicating fewer TE elements in common between the two species. A retroelement (LTR/Ngaro) presented low values of divergence (<10%). The DNA/Kolobok and DNA/hAt-Carlie were the most abundant elements in the most frequent divergence values. When the TEs from *S. senegalensis* chromosome 2 were used to analyze divergence in repetitive elements in syntenic regions of *S. maximus*, the most frequent divergence observed ranged between 9 and 13%, the lowest divergence values obtained in our study. Over the total range, elements DNA/CMC-En, DNA/Maverick, DNA/hAT-Ac and LTR/Gypsy had high coverage. In chromosome 4 analyses, the peaks of divergence were ∼12–14%, with DNA/CMC-EnSpm, DNA/Ginger-1 and LTR/ERV-1 elements presenting higher coverage than other elements.

A LINE (Long interspaced element) element (Rex-Babar) from chromosome 4 presented a high degree of divergence (40%) between *S. senegalensis* and *S. maximus*, indicating a putative strong selection of this retroelement (Table 3, Figure 7d and Figure S19d).

The analysis of TE elements from *S. senegalensis* chromosome 2 and syntenic regions in *O. latipes* revealed the widest spread of divergence values found in this study, with ill-defined peaks of divergence. Figure 7e and Figure S19e show very high coverage peaks, ranging between 23 and 38% (Table 3). The greatest divergence values correspond to two DNA transposon families: DNA/PIF-Harburger and DNA/Tcmar-TC1. In contrast, the DNA/Ginger-1 family showed a null divergence value, indicating high evolutionary conservation between these species and DNA regions. In the TE divergence analysis of *S. senegalensis* chromosome 4 with its syntenic regions of *O. latipes*, the most frequent divergence values were around 20 and 21%, with DNA/CMC-EnSpm and RC Helitron showing the greatest coverage. A second peak with higher, but more discontinuous divergence and lower coverage values was found at 34, 37, and 39% with the most representative families DNA/TcMar-1, Line/Rex-Babar and LTR/ERV. Again, a single family, DNA/Ginger-1, exhibited high coverage and a low divergence value (7%). LINE and SINE (Short interspaced nuclear element) elements throughout this chromosome revealed the greatest degree of divergence (Figure 7f and Figure S19f). Finally, the divergence analysis of TE elements of *D. rerio* syntenic regions with *S. senegalensis* chromosome 2 presented a main peak at 24–27%, with minor peaks around 11–15% (Figure 7g and Figure S19g). The most abundant families for the most frequent divergence values were DNA/Kolobok-T2 and DNA/hAT-Ac. For the minor peaks the most representative families were SINE /tRNA-V and RC/Helitron. A single divergence value of 12% with high coverage was observed for DNA/Kolobok and DNA/hAT-Ac families. For chromosome 4, the most frequent divergence values with the syntenic region of *D. rerio* was around 24–27%. The most abundant families across all divergence peak values were RC/Helitron and a SINE/tRNA-V subfamily (Figure 7h and Figure S19h).

## 3. Discussion

In this work, the evolution of the metacentric chromosome 2 and submetacentric chromosome 4 of *S. senegalensis* has been studied. Synteny and repetitive elements were analyzed in 11 and 10 BACs of chromosomes 2 and 4, respectively. Considering the synteny results for both chromosomes as a whole, BACs map to two main chromosomes in the reference species, indicating that metacentric chromosome 2 and submetacentric chromosome 4 could have been formed by Robertsonian fusions, pericentric inversions and other chromosomal rearrangements. In a previous study, comparable results in the distribution of BACs were observed for chromosome 1 of *S. senegalensis* [2,3,31].

One consequence of these fusions would be the reduction of the number of chromosomes in *S. senegalensis* (2n = 42), from the plesiomorphic condition of 2n = 48 in teleosts. Fish families have followed distinct evolutionary paths in relation to the number of chromosomes. The families *Haemulidae*, *Lutjanidae*, and *Sciaenidae* of the Perciformes order, for example, present remarkable karyotype conservation and the ancestral condition or karyotype stasis is maintained (2n = 48, FN = 48) [32]. In contrast, other orders such as Tetraodontiforms and Gasterosteiforms exhibit reduction in chromosome numbers due to fusion of pairs of ancestral chromosomes [33]. For the family Batrachoididae (Batrachoideforms order), the ancestral condition has been reported to be 2n = 46 instead of 2n = 48, and for the species *Porichthys plectrodon*, the presence of a pair of large metacentric chromosomes in their karyotype suggests a Robertsonian translocation between two acrocentric chromosomes in the evolution of the karyotype toward the actual 2n = 44 [34].

The Pleuronectiformes order shows variation in the number of chromosomes [21]. In addition, species with the same number of chromosomes differ in the FN, including, for example, *C. semilaevis* and *Trinectes inscriptus* (2n = 42). While all chromosomes in *C. semilaevis* are acrocentric, the karyotype of *T. inscriptus* is formed by three large metacentric, one submetacentric and several subtelocentric chromosome pairs. The metacentric chromosomes probably originated from chromosome fusions, while the submetacentric and other subtelocentric pairs originated from pericentric inversions probably from six ancient acrocentric chromosome pairs [14,35]. The karyotype of *S. maximus* (2n = 44) comprises 3 pairs of M/SM and 19 pairs of ST/T chromosomes and differs in one chromosome pair from *S. senegalensis* [36]. These kinds of difference are also observed within the Soleidae family. Hence, *Dagetichthys lusitanica*, has 2n = 42 (FN = 50), with two metacentric, two submetacentric and 17 telocentric chromosome pairs; and *Dicologlossa cuneata*, with 2n = 50 (FN = 54) and with one large metacentric chromosome and several smaller ones, and 23 telocentric pairs. Zoo-FISH studies with these two species indicated that the chromosome 1 of *S. senegalensis* originated from the fusion of two acrocentric chromosomes found in the karyotype of both *D. cuneata* and *D. lusitanicus*. This chromosome pair has been proposed as a proto-sex chromosome in *S. sengalensis* [3]. The results presented in this paper indicate that evolution of chromosomes 2 and 4 depends on the genomic surrounding of TEs that are responsible for the interchange and rearrangement of blocks of DNA.

**Figure 8:**
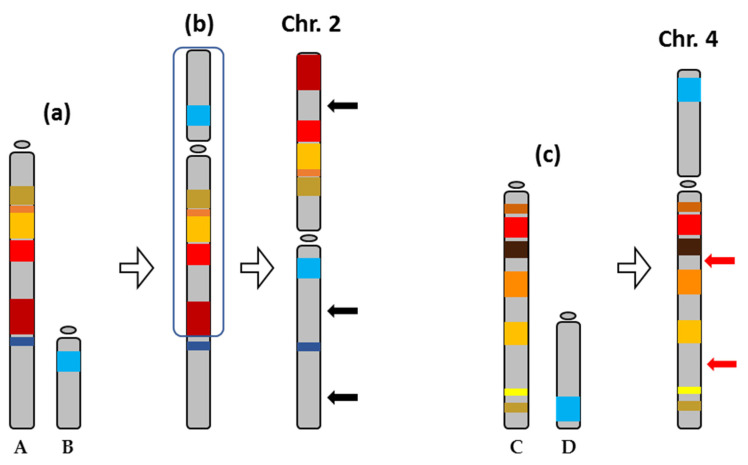
Evolution of the chromosomes 2 and 4 of *Solea senegalensis* from ancestral acrocentric chromosomes, represented as A, B, C, and D. (**a**) A Robertsonian translocation between A and B to originate a new submetacentric chromosome (**b**), which, after pericentric inversion (region inside the outline), formed the metacentric chromosome 2 of *S. senegalensis*. The black arrows indicate probable translocation events that gave rise to the present chromosome 2. (**c**) A Robertsonian translocation between other acrocentric chromosomes, C and D, gave rise to the submetacentric chromosome 4 of *S. senegalensis*. The red arrows indicate possible translocation events that resulted in the present chromosome 4.

The transposable elements analysis of *S. senegalensis* chromosomes 2 and 4 measured the abundance of different TE classes. Chromosome 2 showed a greater abundance of class II elements (DNA transposons), in terms of NL/Mb and coverage, than chromosome 4. In most fish genomes, Class II DNA transposons are the most abundant component [5,37], although many TE superfamilies are present in this group of organisms, presenting evidence of greater diversity than in other vertebrates [8]. Among TE families, Tc/mariner, hAT, L1, L2, and Gypsy are the most widespread and predominant TE superfamilies in Actinopterygian genomes [38,39]. However, some organisms present a predominance of specific TE superfamilies [5]; thus they could have played a pivotal role in their evolution. The TE abundance observed in chromosome 2 (Table 2) might have facilitated the multiple chromosomal rearrangements, such as pericentric inversions, leading to the formation of this chromosome (Figure 8) [14,31].

The analysis of two *S. senegalensis* chromosomes has shown the differences between them in the presence of classes and families of TEs. Retroviral LTR elements, hobo-Activator and Tc1-Pogo DNA transposons and LINE elements such as RTE/Bov-B and L1/CIN4 LINE showed 2-to-9 fold differences, in terms of NL/Mb and coverage. These findings indicate that these TEs could play a main role in their differentiation and evolution. Moreover, the TE analysis per mapped BACs, on various chromosomes, has allowed us to analyze the distribution on specific chromosome arms. The results show a general pattern of high TEs abundance (measured as NL/Mb and coverage) next to telomeric and centromeric regions, as described previously for *S. sengalensis* chromosome 1 [9]. However one BAC (46P22) located at an interstitial position on chromosome 4 presented a high degree of abundance of TEs, when compared with other BACs analyzed in this and in a previous *S. senegalensis* study [9]. It would be important to note that none of the studied BAC was located in the centromere of the chromosomes, as centromeres are known to be rich in repeat sequences further studies including them could complete data shown in this paper.

The comparative mapping net plot revealed an isolated node cluster for this BAC, reflecting a different evolution process in that BAC region. The syntenic regions of this BAC in other fishes are always found in telomeric locations, so this chromosome could have kept its abundance of TEs during evolution. High TE abundance in these chromosomes could also be associated with selection events such as in the evolution of sex chromosomes, as found in *S. senegalensis* chromosome 1, where TEs could account for the evolution of the putative sex-determining chromosome of this species, although sex chromosomes evolve differently than autosomes [2]. The number of Single Sequence Repeats (SSR) loci was in the range found in previous *S. senegalensis* studies ∼400–1000 NL/Mb [9], except for telomeric BACs in chromosome 4, which displayed higher values (1000–1700 NL/Mb). The SSR coverage in these two chromosomes (1.5–2.2%) was slightly higher than in other chromosomes, as found in a previous analysis of *S. senegalensis* [9]. These values are slightly lower than those in the green puffer fish *T. nigroviridis*, where SSRs account for 3.21% of the genome [40] but higher than that found in the genome of the fugu puffer fish *T. rubripes* (1.29%) [41]. The relationship between meiotic recombination and TEs has been discussed recently [42], and its role in the coverage and distribution of TEs in particular chromosome regions of *S. senegalensis* could provide insights into their genome dynamics and evolution.

In order to estimate divergence and “age” history of TEs for the syntenic regions of *S. senegalensis* chromosomes 2 and 4 and those of four other fish species, Kimura distances were calculated for all TE copies. Divergence is correlated with the age of the activity [8], where low K-values (similar TEs) are indicative of more recent activity (left side of the graphics), while high K-values (divergent TEs) have been created by more ancient transposition events (right side of the graphics). All syntenic regions analyzed in the other fish species, for both chromosomes 2 and 4, have been strongly shaped by DNA transposons (Class II), except for syntenic regions of chromosome 2 in *S. maximus*, which presents the most amplifications of LTR elements (Class I), with a major and recent burst of activity (∼K-value 10) (Figure S19). In contrast, ancient amplifications of elements (K-value around 37) in LINEs in the syntenic region in *O. latipes* for *S. senegalensis* chromosome 4 was observed. Syntenic regions on chromosomes 2 and 4 showed a major burst of activity for more recent copies in *S. maximus*, and a more ancient amplification event in the other analyzed flatfish *C. semilaevis*, in relation to *S. senegalensis*. In chromosome 2 *D. rerio* revealed two major bursts of activity with ancient copies predominant but fewer recent copies (Figure S19). In teleosts as a whole, significant interspecific differences in TE divergence have been observed [43], generally with one or two bursts of transposition [5,8]. Teleost genomes generally contain fewer ancient copies (K-values >25) than the genomes of other organisms such as mammals, suggesting differences in the process of elimination [8]. All these data show a differentiation in the divergence values of TE elements for both chromosomes in comparison with other teleosts, revealing this method as efficient and useful for future analyses of the evolution of transposable elements in the genome of soles.

This paper provides data about karyotype evolution in Pleuronectiformes, for which cytogenomics information is scarce. We conclude that chromosomes 2 and 4 of *S. senegalensis* evolved by Robertsonian fusion accompanied by some additional rearrangements that could be mediated by TEs, given the high number found in the studied sequences. A genome comparison showed more similarities between *S. senegalensis* and *C. semilaevis* than with *S. maximus*. However, synteny results suggested otherwise, indicating that the evolution of the S. senegalensis karyotype has been notably different from those other species.

## 4. Materials and Methods

### 4.1. BAC Clones

The 21 BAC clones used in this study were obtained from a BAC library of *S. senegalensis* comprised of 29,184 clones distributed in 384–well plates (76 plates in total). The BAC screening was carried out using a 4D–PCR method [44] as described by García-Cegarra et al. [30]. BACs were named according to the plate library number and their row (A–P) and column (1–24) coordinates. The BAC clones are available in the GenBank database under the following accession numbers: AC278120.1 (BAC 4D15), AC278094.1 (BAC 36I3), AC270104.1 (BAC 6P22), AC278095.1 (BAC 38N10), AC278071.1 (BAC 46C5), AC278079.1 (BAC 52G10), AC278093.1 (BAC 60P19), AC270102.1 (BAC 21O23), MW199155 (BAC 9E8), MW199152 (BAC 3F15), MW199159 (BAC 19L16), AC278070.1 (BAC 3C15), AC278106.1 (BAC 8A23), AC278101.1 (12D24), AC278098.1 (BAC 36H2), AC278053.1 (BAC 36H3), AC278110.1 (BAC 36J2), AC278074.1 (BAC 46B2), AC278065.1 (BAC 46P22), AC270101.1 (BAC12N15), AC275287.1 (BAC 30J4).

### 4.2. Double FISH-BAC

Chromosome preparations were carried out as described by Rodriguez et al. [2], using larvae (1–3 days after hatching) of *S. senegalensis*. The DNA-BAC was purified using Plasmid Midi Kit (Quiagen, Hilden, Germany) following manufacturer’s instructions, and then labelled using Biotin or Digoxigenin Nick Translation Mix (Roche Molecular Biochemical), as described by manufacturer’s instructions. Pre-treatment of chromosome preparations and hybridization were carried out following the protocol described by García-Cegarra et al. [30]. For the immunocytochemistry detection, the antibodies described in Rodriguez et al. [2] were used. The antibodies were prepared in Tween Non-Fat Milk (TNFM, 4x SSC, 0.05% Tween 20.5% skim milk). Chromosome staining was carried out with 0.4 mg/mL of 4’,6-diamidino-2-phenylindole DAPI-Vectashield (Antifade Mounting Medium) (Vector), and the images were examined with a Zeiss Palm MicroBeam microdissector and fluorescence microscopes equipped with an AxioCam MRm digital camera.

### 4.3. Sequencing and Synteny Analysis

BAC DNA was purified using the Large Construct kit (Quiagen, Hilden, Germany) and then sequenced using the MiSeq Illumina sequencing platform (Illumina, San Diego, CA, USA). BAC annotation was performed as described in García-Angulo et al. [3].

For micro-synteny analysis, the program Geneious R11 [45] was used to order the genes within each contig of *S. senegalensis* and to estimate the distance between them. The order of the contigs in each BAC was obtained using the *Ensembl* database and *Cynoglossus semilaevis* genome as reference.

For synteny studies, the comparative genomic analysis was carried out using eight species of fish: *C. semilaevis* (2n = 42), *Scophthalmus maximus* (2n = 44), *Sparus aurata* (2n = 48), *Gasterosteus aculeatus* (2n = 42), *Xiphophorus maculatus* (2n = 48), *Oryzias latipes* (2n = 48), *Danio rerio* (2n = 50), and *Lepisosteus oculatus* (2n = 58 [46]). The *Ensembl* database was used to compare the gene sequences of *S. senegalensis* with the reference species.

#### Synteny Relationship Net-Graphs

The correspondence between syntenic regions of *S. senegalensis* and the other fish species was represented using nodes (BACs and fish chromosomes) and edges (number of shared positions) with the igraph package v1.2.5 in R [47] considering only syntenic relationships. The force-directed Fruchterman–Reingold layout algorithm was used to place vertices on the plane [48]. The main parameters used were: niter = 10,000, start-temp = sqrt (vcount (graph)), where niter is the number of iterations to perform, start.temp is the maximum amount of movement allowed along one axis, within one step, for a vertex (it is decreased linearly to zero during the iteration) and vcount the vertex number.

### 4.4. Repetitive Elements Analysis

After BAC clone mapping, a statistical analysis of repetitive elements was carried out using a homology-based approach with the Repbase database (release 23.07) and Repeat Masker software v.4.0.9 (from now on RM) [49]. The repetitive elements analyzed were: DNA retrotransposons, retroelements, low complexity, simple repeats, and satellite sequences. The low complexity elements and DNA satellite coverage was measured as the quantity of sequences (bp) per BAC sequences length analyzed (%), and the average number of TEs was calculated, in relation to the BAC sequences length, as the total number of identified loci per Mb.

### 4.5. Transposable Elements Divergence

After pooling *S. senegalensis* BAC clone sequences per chromosome (Chromosomes 2 and 4), repetitive elements were first identified using RM with the *D. rerio* RepBase repeat library. Low-complexity repeats were ignored (-nolow) and a sensitive (-s) search was performed. From RM results, *S. senegalensis* repeat libraries, one per chromosome, were then constructed using home-made scripts and bedtools software v2.25.0 [50]. Sequence Dereplicator and Database Curator python software was used to dereplicate redundant sequences (https://github.com/Eslam-Samir-Ragab/Sequence-database-curator). Subsequently, syntenic regions from *C. semilaevis*, *S. maximus*, *O. latipes* and *D. rerio* species were mined from the Ensemble database, and the *S. senegalensis* repeat element libraries were used to identify repetitive elements and their divergences with RM.

A Kimura distance-based copy divergence analysis relative to the *S. senegalensis* TE elements database made per chromosomes (2 and 4) and four fish species, from the closer *C. semilaevis* and *S. maximus*, to the more distant species *O. latipes* and *D. rerio*, was carried out.

Perl scripts were used to calculate divergence analytic measures on the RM alignment files and to create a Repeat Landscape graph using the divergence summary data (https://github.com/rmhubley/RepeatMasker). Results were analyzed per family and they were also grouped for the four different types of TEs (DNA transposons, LTR, LINE, and SINE retrotransposons) [8]. 

## Figures and Tables

**Table 1:** BAC clones analyzed in this study and gene annotation.

Chromosome	BAC	Gene Annotation	References
**Chr. 2**	52G10	*lamb1*, *ssbp1*, *creb3l2*, *dgki*, *calu*, *opn1sw*, *tnpo3*, *irf5*, *atp6v1f*, *dennd6b*, *gcc1*, *arf5*, *lta4h*, *mical3a*	[3,9]
	6P22	*mri1*, *rfx1*, *dcaf15*, *khsrp*, *slc25a24*, *rgs5*, *c19orf53*, *gng10*, *ssx2ip*, *nanos3*, *cc2d1a*, *umod*, *slc26a11*, *sgsh*, *npb*, *sirt7*, *pcyt2*, *gnao1*, *mafg*, *map2k5*, *skor1b*	[29]
	9E8	*thrβ*, *nr1d2*, *ube2e2*	[4,30]
	60P19	*trim39*, *c1qtnf3*, *casq1b*, *pea15*, *mrpl48*, *supt16h*, *trim25*, *fam234a*, *mrc2*	[3,4]
	46C5	*ndrg2*, *hnrnpc*, *fhod1*, *klhl33*, *c1ql2*, *parp14*, *mrc1*, *slc12a3*, *ltb4r*, *myadm*, *arhgef11a*, *apol6*	[3]
	36I3	*chd3*, *tnfsf12*	[3]
	4D15	*ppme1*, *rnf150*, *inpp4b*, *hmgb2*, *dlg4*	[3,9]
	38N10	*tmbim4*, *b2m*, *gdi2*, *asb13*, *net1*, *hspa14*, *manf*, *irak3*, *prl*	[3]
	3F15	*slc18a3a*, *rgr*, *lrit1*	This work
	21O23	*fabp2*, *dmrt4*, *adcyap1r1*	[29]
	19L16	*kiaa1324*, *c1orf194*, *smc5*, *fam107b*	This work
**Chr. 4**	12N15	*tuba1c*, *taar13c*, *atp1b1b*, *ube3a*, *gpd2*, *hsf2bp*, *cnga3*, *ddx4*, *ccdc14*, *ankrd10*, *dpp10*, *nr4a2*	[3,29]
	3C15	*pde11a*, *cycsb*, *osbpl6*	[3]
	46B2	*uggt2*, *dnajc3*, *cldn10*, *abcc4*, *dct*, *sox21*, *gpc6*	[3]
	30J4	*nlrc3*, *wdr90*, *rhot2*, *H1.0B*, *rhbdl1*, *wdr24*, *anks3*, *c8orf33*, *H3.3*, *gcgr*, *pcdh8*, *ednrb*, *cog3*, *mid1*, *arhgap6*, *tlr7*, *tlr8*, *tyb12*, *egfl6*	[3,30,31]
	12D24	*igsf3*, *rpe*, *ackr3*, *nqo1*, *nhlh2*, *vangl1*, *casq2*, *kcne2*, *slc5a3*, *dop1b*, *morc3*, *ptprn*	[3,4]
	8A23	*hibch*, *c2orf88*, *mstn*, *pms1*, *ormdl1*, *adat3*, *alkbh6*, *osgepl1*, *fkbp7*, *gls*	[3]
	46P22	*zfpm1*, *trhr2*	[4]
	36J2	*crygs*	[3]
	36H3, 36H2	*ccdc141*, *tchh*, *ttn*	[3]

**Table 2:** Summary of repeat types present in the chromosomes 2 and 4 of *Solea senegalensis.*

	NL/Mb		Coverage (%)	
Class/Family	Chr.2	Chr.4	Chr.2	Chr.4
Retroelements	114.209	124.649	1.12	1.68
SINEs:	34.686	22.879	0.34	0.23
Penelope	1.692	3.156	0.01	0.03
LINEs:	47.376	44.968	0.57	0.81
L2/CR1/Rex	27.072	29.190	0.32	0.58
R1/LOA/Jockey	3.384	3.156	0.02	0.03
R2/R4/NeSL	0.000	0.789	0	0.11
RTE/Bov-B	9.306	0.789	0.14	0.02
L1/CIN4	2.538	4.734	0.02	0.03
LTR elements	32.148	56.802	0.21	0.64
BEL/Pao	5.922	4.734	0.04	0.03
Ty1/Copia	0.000	0.000	0	0
Gypsy/DIRS1	13.536	22.090	0.08	0.29
Retroviral	3.384	20.512	0.02	0.24
DNA transposons	317.247	268.232	2.97	2.81
hobo-Activator	141.281	85.203	1.15	0.69
Tc1-IS630-Pogo	45.684	20.512	0.83	0.43
PiggyBac	1.692	1.578	0.02	0.04
Tourist/Harbinger	9.306	7.100	0.16	0.07
Other	0.846	0.000	0	0

**Table 3:** Summary of TEs divergence analysis carried out between syntenic regions of *Cynoglossus semilaevis, Scophthalmus maximus, Oryzias latipes,* and *Danio rerio* with *Solea senegalensis* in chromosomes 2 and 4.

Chromosome of *S. senegalensis*.	Spp.	Main Divergence Peak (K-Value)	Families
2	*C. semilaevis*	16–21	DNA/CMC/Emspm, DNA/Kolobok and DNA/hAT-Ac elements
	*S. maximus*	9–13	DNA/CMC-En, DNA/Maverick, DNA/hAT-Ac and LTR/Gypsy
	*O. latipes*	23–38	DNA/PIF-Harburger and DNA/Tcmar-TC1
	*D. rerio*	24–27	DNA/Kolobok-T2
4	*C. semilaevis*	22–27	DNA/Kolobok and DNA/hAt-Carlie
	*S. maximus*	12–14	DNA/CMC-EnSpm, DNA/Ginger-1 and LTR/ERV-1
	*O. latipes*	20–21	DNA/CMC-EnSpm and RC Helitron
	*D. rerio*	24–27	RC/Helitron and SINE/tRNA-V

## Data Availability

https://www.ncbi.nlm.nih.gov/genbank/.

## Supplementary Materials

Supplementary materials can be found at https://www.mdpi.com/1422-0067/22/4/1614/s1.

## References

[1] Symonová R., Howell W.M. (2018). Vertebrate Genome Evolution in the Light of Fish Cytogenomics and rDNAomics. Genes.

[2] Rodríguez M.E., Molina B., Merlo M.A., Arias-Pérez A., Portela-Bens S., García-Angulo A., Cross I., Liehr T., Rebordinos L. (2019). Evolution of the proto sex-chromosome in Solea senegalensis. Int. J. Mol. Sci..

[3] García-Angulo A., Merlo M.A., Portela-Bens S., Rodríguez M.E., García E., Al-Rikabi A.B.H., Liehr T., Rebordinos L. (2018). Evidence for a Robertsonian fusion in Solea senegalensis (Kaup, 1858) revealed by zoo-FISH and comparative genome analysis. BMC Genom..

[4] García-Angulo A., Merlo M.A., Rodríguez M.E., Portela-Bens S., Liehr T., Rebordinos L. (2019). Genome and Phylogenetic Analysis of Genes Involved in the Immune System of Solea senegalensis – Potential Applications in Aquaculture. Front. Genet..

[5] Shao F., Han M., Peng Z. (2019). Evolution and diversity of transposable elements in fish genomes. Sci. Rep..

[6] Jurka J., Kapitonov V., Pavlicek A., Klonowski P., Kohany O., Walichiewicz J. (2005). Repbase Update, a database of eukaryotic repetitive elements. Cytogenet. Genome Res..

[7] Manchado M., Planas J.V., Cousin X., Rebordinos L., Claros M.G. (2019). Genetic and Genomic Characterization of Soles. Biol. Sole.

[8] Chalopin D., Naville M., Plard F., Galiana D., Volff J.-N. (2015). Comparative Analysis of Transposable Elements Highlights Mobilome Diversity and Evolution in Vertebrates. Genome Biol. Evol..

[9] García E., Cross I., Portela-Bens S., Rodríguez M.E., García-Angulo A., Molina B., Cuadrado Á., Liehr T., Rebordinos L. (2019). Integrative genetic map of repetitive DNA in the sole Solea senegalensis genome shows a Rex transposon located in a proto-sex chromosome. Sci. Rep..

[10] Nelson J.S., Grande T.C., Wilson M.V.H. (2016). Fishes of the World.

[11] Glasauer S.M.K., Neuhauss S.C. (2014). Whole-genome duplication in teleost fishes and its evolutionary consequences. Mol. Genet. Genom..

[12] Ohno S. (1970). Evolution by Gene Duplication.

[13] Brum M.J.L., Galetti P.M. (1997). Teleostei ground plan karyotype. J. Comp. Biol..

[14] Bitencourt J.A., Sampaio I., Ramos R.T., Affonso P.R. (2014). Chromosomal fusion in Brazilian populations of Trinectes inscriptus Gosse, 1851 (Pleuronectiformes; Achiridae) as revealed by internal telomere sequences (ITS). J. Exp. Mar. Biol. Ecol..

[15] Shi W., Chen S., Kong X., Si L., Gong L., Zhang Y., Yu H. (2018). Flatfish monophyly refereed by the relationship of Psettodes in Carangimorphariae. BMC Genom..

[16] Campbell M.A., Chen W.-J., Lopez J.A. (2013). Are flatfishes (Pleuronectiformes) monophyletic?. Mol. Phylogenet. Evol..

[17] Campbell M.A., Chen W.-J., López J.A. (2014). Molecular data do not provide unambiguous support for the monophyly of flatfishes (Pleuronectiformes): A reply to Betancur-R and Ortí. Mol. Phylogenetics Evol..

[18] Campbell M.A., López J.A., Satoh T.P., Chen W.-J., Miya M. (2014). Mitochondrial genomic investigation of flatfish monophyly. Gene.

[19] Harrington R.C., Faircloth B.C., Eytan R.I., Smith W.L., Near T.J., Alfaro M.E., Friedman M. (2016). Phylogenomic analysis of carangimorph fishes reveals flatfish asymmetry arose in a blink of the evolutionary eye. BMC Evol. Biol..

[20] Hinegardner R. (1968). Evolution of Cellular DNA Content in Teleost Fishes. Am. Nat..

[21] Azevedo M.F.C., Oliveira C., Pardo B.G., Martínez P., Foresti F. (2007). Cytogenetic characterization of six species of flatfishes with comments to karyotype differentiation patterns in Pleuronectiformes (Teleostei). J. Fish Biol..

[22] Chen S., Zhang G., Shao C., Huang Q., Liu G., Zhang P., Song W., An N., Chalopin D., Volff J.-N. (2014). Whole-genome sequence of a flatfish provides insights into ZW sex chromosome evolution and adaptation to a benthic lifestyle. Nat. Genet..

[23] Howe K., Clark M.D., Torroja C.F., Torrance J., Berthelot C., Muffato M., Collins J.E., Humphray S., McLaren K., Matthews L. (2013). The zebrafish reference genome sequence and its relationship to the human genome. Nature.

[24] Hardie D.C., Hebert P.D. (2004). Genome-size evolution in fishes. Can. J. Fish. Aquat. Sci..

[25] Pardo B.G., Bouza C., Castro J., Martínez P., Sánchez L. (2001). Localization of ribosomal genes in Pleuronectiformes using Ag-, CMA3-banding and in situ hybridization. Heredity.

[26] Robledo D., Hermida M., Rubiolo J.A., Fernández C., Blanco A., Bouza C., Martínez P. (2017). Integrating genomic resources offlatfish (Pleuronectiformes) to boost aquaculture production. Comp. Biochem. Physiol. Part D Genom. Proteom..

[27] Vega L., Díaz E., Cross I., Rebordinos L. (2002). Caracterizaciones citogenética e isoenzimática del lengauado Solea senegalensis Kaup, 1858. Bol. Inst. Esp. Oceanogr..

[28] Cross I., Merlo A., Manchado M., Infante C., Cañavate J.-P., Rebordinos L., Torres M.A.M. (2006). Cytogenetic characterization of the sole Solea senegalensis (Teleostei: Pleuronectiformes: Soleidae): Ag-NOR, (GATA) n, (TTAGGG) n and ribosomal genes by one-color and two-color FISH. Genetics.

[29] Portela-Bens S., Merlo M.A., Rodríguez M.E., Cross I., Manchado M., Kosyakova N., Liehr T., Rebordinos L. (2016). Integrated gene mapping and synteny studies give insights into the evolution of a sex proto-chromosome in Solea senegalensis. Chromosom.

[30] Cegarra A.M.G., Merlo M., Ponce M., Portela-Bens S., Cross I., Manchado M., Rebordinos L. (2013). A Preliminary Genetic Map inSolea senegalensis(Pleuronectiformes, Soleidae) Using BAC-FISH and Next-Generation Sequencing. Cytogenet. Genome Res..

[31] Merlo M.A., Iziga R., Portela-Bens S., Cross I., Kosyakova N., Liehr T., Manchado M., Rebordinos L. (2017). Analysis of the histone cluster in Senegalese sole (Solea senegalensis): Evidence for a divergent evolution of two canonical histone clusters. Genome.

[32] Da Motta-Neto C.C., Cioffi M.D.B., Da Costa G.W.W.F., Amorim K.D.J., Bertollo L.A.C., Artoni R.F., Molina W.F. (2019). Overview on Karyotype Stasis in Atlantic Grunts (Eupercaria, Haemulidae) and the Evolutionary Extensions for Other Marine Fish Groups. Front. Mar. Sci..

[33] Amores A., Catchen J., Nanda I., Warren W., Walter R., Schartl M., Postlethwait J.H. (2014). A RAD-Tag Genetic Map for the Platyfish (Xiphophorus maculatus) Reveals Mechanisms of Karyotype Evolution Among Teleost Fish. Genetics.

[34] Úbeda-Manzanaro M., Merlo M.A., Palazón J.L., Cross I., Sarasquete C., Rebordinos L. (2010). Chromosomal mapping of the major and minor ribosomal genes, (GATA)n and U2 snRNA gene by double-colour FISH in species of the Batrachoididae family. Genetics.

[35] Sun A., Chen S.-L., Gao F.-T., Li H.-L., Liu X.-F., Wang N., Sha Z.-X. (2015). Establishment and characterization of a gonad cell line from half-smooth tongue sole Cynoglossus semilaevis pseudomale. Fish Physiol. Biochem..

[36] Taboada X., Pansonato-Alves J.C., Foresti F., Martínez P., Viñas A., Pardo B.G., Bouza C. (2014). Consolidation of the genetic and cytogenetic maps of turbot (Scophthalmus maximus) using FISH with BAC clones. Chromosoma.

[37] Chalopin D., Volff J.-N. (2017). Analysis of the spotted gar genome suggests absence of causative link between ancestral genome duplication and transposable element diversification in teleost fish. J. Exp. Zool. Part B Mol. Dev. Evol..

[38] Gao B., Shen D., Xue S., Chen C., Cui H., Song C. (2016). The contribution of transposable elements to size variations between four teleost genomes. Mob. DNA.

[39] Yuan Z., Liu S., Zhou T., Tian C., Bao L., Dunham R.A., Liu Z. (2018). Comparative genome analysis of 52 fish species suggests differential associations of repetitive elements with their living aquatic environments. BMC Genom..

[40] Chistiakov D.A., Hellemans B., Volckaert F.A. (2006). Microsatellites and their genomic distribution, evolution, function and applications: A review with special reference to fish genetics. Aquaculture.

[41] Edwards Y.J., Elgar G., Clark M.S., Bishop M.J. (1998). The identification and characterization of microsatellites in the compact genome of the japanese pufferfish, Fugu rubripes: Perspectives in functional and comparative genomic analyses. J. Mol. Biol..

[42] Underwood C.J., Choi K. (2019). Heterogeneous transposable elements as silencers, enhancers and targets of meiotic recombination. Chromosoma.

[43] Carducci F., Barucca M., Canapa A., Carotti E., Biscotti M.A. (2020). Mobile Elements in Ray-Finned Fish Genomes. Life.

[44] Asakawa S., Abe I., Kudoh Y., Kishi N., Wang Y., Kubota R., Kudoh J., Kawasaki K., Minoshima S., Shimizu N. (1997). Human BAC library: Construction and rapid screening. Gene.

[45] Kearse M., Moir R., Wilson A., Stones-Havas S., Cheung M., Sturrock S., Buxton S., Cooper A., Markowitz S., Duran C. (2012). Geneious Basic: An integrated and extendable desktop software platform for the organization and analysis of sequence data. Bioinformatics.

[46] Braasch I., Gehrke A.R., Smith J.J., Kawasaki K., Manousaki T., Pasquier J., Amores A., Desvignes T., Batzel P., Catchen J. (2016). The spotted gar genome illuminates vertebrate evolution and facilitates human-teleost comparisons. Nat. Genet..

[47] Csardi G., Nepusz T. (2006). The igraph software package from complex network research. Int. J. Complex Syst..

[48] Fruchterman T.M.J., Reingold E.M. (1991). Graph drawing by force-directed placement. Software Pr. Exp..

[49] Smit A.F.A., Hubley R., Green P. RepeatMasker Open-3.0 (1996–2010). http://www.repeatmasker.org/.

[50] Quinian A.R., Hall I.M. (2010). BEDTools: A flexible suite of utilities for comparing genomic features. Bioinformatics.

