# Supplementary material for: Cytogenomics Unveil Possible Transposable Elements Driving Rearrangements in Chromosomes 2 and 4 of Solea senegalensis
Source: Int J Mol Sci. 2021 Feb 5;22(4):1614. doi: 10.3390/ijms22041614 (PMC7915175; doi:10.3390/ijms22041614)
Supplement: Supplementary file 1 [file ijms-22-01614-s001.zip › Supplementary Materials Legends.docx]

**Supplementary Materials Legends**

**Table S1.** Summary of repeat types present in BAC sequences located in the chromosome 2 of *Solea senegalensis*. Coverage measured as percentage of repeat elements per BAC length.

**Table S2.** Summary of repeat types present in BAC sequences located in the chromosome 2 of *Solea senegalensis*. NL/Mb = Number of loci per Mb of BAC sequenced.

**Table S3.** Summary of repeat types present in BAC sequences located in the chromosome 4 of *Solea senegalensis*. Coverage measured as percentage of repeat elements per BAC length.

**Table S4.** Summary of repeat types present in BAC sequences located in the chromosome 4 of *Solea senegalensis*. NL/Mb = Number of loci per Mb of BAC sequenced.

**Figure S1**. Syntenic map of the metacentric chromosome 2 of *Solea senegalensis* with respect to *Cynoglossus semilaevis*. The % of the genes of BACs found in each chromosome of *C. semilaevis* is shown between parentheses. This % was calculated taking into account the total number of the genes (85 genes) for chromosome 2 of *S. senegalensis*.

Legend symbols: *Unordered genes in the analyzed BAC of *S. senegalensis*. ^+^Genes with low values in BLASTp analysis with *C. semilaevis.*

**Figure S2**. Syntenic map of the metacentric chromosome 2 of *Solea senegalensis* with respect to *Scophthalmus maximus*. The % of the genes of BACs found in each chromosome of *S. maximus* is shown between parentheses. This % was calculated taking into account the total number of the genes (85 genes) for chromosome 2 of *S. senegalensis*.

Legend symbols: *Unordered genes in the analyzed BAC of *S. senegalensis*. ^+^Gene with low values in BLASTp analysis with *S. maximus.* ^✦^Genes found in chromosome 19 of *S. maximus*. ^✧^Gene found in chromosome 18 of *S. maximus*.

**Figure S3**. Syntenic map of the metacentric chromosome 2 of *Solea senegalensis* with respect to *Sparus aurata.* The % of the genes of BACs found in each chromosome of *S. aurata* is shown between parentheses. This % was calculated taking into account the total number of the genes (85 genes) for chromosome 2 of *S. senegalensis*.

Legend symbols: *Unordered genes in the analyzed BAC of *S. senegalensis*. ^+^Genes with low values in BLASTp analysis with *S. aurata.* ^✞^Genes found in chromosome 4 of *S. aurata*. ^✧^Gene found in chromosome 13 of *S. aurata*. ^✠^Gene found in chromosome 6 of *S. aurata*. ^✟^Gene found in chromosome 21 of *S. aurata.*

**Figure S4**. Syntenic map of the metacentric chromosome 2 of *Solea senegalensis* with respect to *Gasterosteus aculeatus.* The % of the genes of BACs found in each chromosome of *G. aculeatus* is shown between parentheses. This % was calculated taking into account the total number of the genes (85 genes) for chromosome 2 of *S. senegalensis*.

Legend symbols: *Unordered genes in the analyzed BAC of *S. senegalensis.* ^+^Genes with low values in BLASTp analysis with *G. aculeatus*. ^✦^Genes found in chromosome II of *G. aculeatus*. ^✧^Gene found in chromosome V of *G. aculeatus*.

**Figure S5**. Syntenic map of the metacentric chromosome 2 of *Solea senegalensis* with respect to *Xiphophorus maculatus.* The % of the genes of BACs found in each chromosome of *X. maculatus* is shown between parentheses. This % was calculated taking into account the total number of the genes (85 genes) for chromosome 2 of *S. senegalensis*.

Legend symbols: *Unordered genes in the analyzed BAC of *S. senegalensis*. ^+^Genes with low values in BLASTp analysis with *X. maculatus.* ^✦^Genes found in chromosome 4 of *X. maculatus*.

**Figure S6**. Syntenic map of the metacentric chromosome 2 of *Solea senegalensis* with respect to *Oryzias latipes.* The % of the genes of BACs found in each chromosome of *O. latipes* is shown between parentheses. This % was calculated taking into account the total number of the genes (85 genes) for chromosome 2 of *S. senegalensis*.

Legend symbols: *Unordered genes in the analyzed BAC of *S. senegalensis*. ^+^Genes with low values in BLASTp analysis with *O. latipes*. ^✧^Genes found in chromosome 3 of *O. latipes*. ^✦^Gene found in chromosome 19 of *O. latipes*. ^✠^Gene found in chromosome 8 of *O. latipes*. ^✞^Gene found in chromosome 14 of *O. latipes*.

**Figure S7**. Syntenic map of the metacentric chromosome 2 of *Solea senegalensis* with respect to *Danio rerio*. The % of the genes of BACs found in each chromosome of *D. rerio* is shown between parentheses. This % was calculated taking into account the total number of the genes (85 genes) for chromosome 2 of *S. senegalensis*.

Legend symbols: *Unordered genes in the analyzed BAC of *S. senegalensis.* ^+^Genes with low values in BLASTp analysis with *D. rerio*. ^✦^Gene found in chromosome 10 of *D. rerio*. ^✧^Gene found in chromosome 24 of *D. rerio*. ^✠^Gene found in chromosome 11 of *D. rerio*. ^✞^ Genes found in chromosome 7 of *D. rerio*. ^✟^ Gene found in chromosome 21 of *D. rerio*. ^☨^ Gene found in chromosome 5 of *D. rerio*.

**Figure S8.** Syntenic map of the metacentric chromosome 2 of *Solea senegalensis* with respect to *Lepisosteus oculatus*. The % of the genes of BACs found in each chromosome of *L. oculatus* is shown between parentheses. This % was calculated taking into account the total number of the genes (85 genes) for chromosome 2 of *S. senegalensis*.

Legend symbols: *Unordered genes in the analyzed BAC of *S. senegalensis.* ^+^Genes with low values in BLASTp analysis with *L. oculatus*.^✦^Genes found in chromosome LG3 of *L. oculatus*. ^✧^Gene found in chromosome LG2 of *L. oculatus*. ^✠^Gene found in chromosome LG23 of *L. oculatus*.

**Figure S9.** Taxonomic tree with the species of fishes used in this study. For manual construction of the tree, the information was obtained from NCBI database (https://www.ncbi.nlm.nih.gov/guide/taxonomy/).

**Figure S10**. Syntenic map of the submetacentric chromosome 4 of *Solea senegalensis* respect to *Cynoglossus semilaevis.* The % of the genes of BACs found in each chromosome of *C. semilaevis* is shown between parentheses. This % was calculated taking into account the total number of the genes (69 genes) for chromosome 4 of *S. senegalensis*.

Legend symbols: ^+^Genes with low values in BLASTp analysis with *C. semilaevis*.

**Figure S11**. Syntenic map of the submetacentric chromosome 4 of *Solea senegalensis* respect to *Scophthalmus maximus.* The % of the genes of BACs found in each chromosome of *S. maximus* is shown between parentheses. This % was calculated taking into account the total number of the genes (69 genes) for chromosome 4 of *S. senegalensis*.

Legend symbols: ^+^Gene with low values in BLASTp analysis with *S. maximus*.

**Figure S12**. Syntenic map of the submetacentric chromosome 4 of *Solea senegalensis* with respect to *Sparus aurata.* The % of the genes of BACs found in each chromosome of *S. aurata* is shown between parentheses. This % was calculated taking into account the total number of the genes (69 genes) for chromosome 4 of *S. senegalensis*.

**Figure S13**. Syntenic map of the submetacentric chromosome 4 of *Solea senegalensis* with respect to *Gasterosteus aculeatus.* The % of the genes of BACs found in each chromosome of *G. aculeatus* is shown between parentheses. This % was calculated taking into account the total number of the genes (69 genes) for chromosome 4 of *S. senegalensis*.

Legend symbols: ^+^Genes with low values in BLASTp analysis with *G. aculeatus*. ^✠^Gene found in chromosome VIII of *G. aculeatus*. ^✟^Genes located in a different position in chromosome XVI of *G. aculeatus*.

**Figure S14**. Syntenic map of the submetacentric chromosome 4 of *Solea senegalensis* with respect to *Xiphophorus maculatus.* The % of the genes of BACs found in each chromosome of *X. maculatus* is shown between parentheses. This % was calculated taking into account the total number of the genes (69 genes) for chromosome 4 of *S. senegalensis*.

Legend symbols: ^+^Genes with low values in BLASTp analysis with *X. maculatus*.

**Figure S15**. Syntenic map of the submetacentric chromosome 4 of *Solea senegalensis* with respect to *Oryzias latipes.* The % of the genes of BACs found in each chromosome of *O. latipes* is shown between parentheses. This % was calculated taking into account the total number of the genes (69 genes) for chromosome 4 of *S. senegalensis*.

Legend symbols: ^+^Genes with low values in BLASTp analysis with *O. latipes.* **^✦^**Gene found in other position different to the rest of the genes in the chromosome 21 in *O. latipes*.

**Figure S16**. Syntenic map of the submetacentric chromosome 4 of *Solea senegalensis* with respect to *Danio rerio.* The % of the genes of BACs found in each chromosome of *D. rerio* is shown between parentheses. This % was calculated taking into account the total number of the genes (69 genes) for chromosome 4 of *S. senegalensis*.

Legend symbols: ^+^Genes with low values in BLASTp analysis with *D. rerio.* ^✦^Gene found in chromosome 23 of *D. rerio*. ^✧^Gene found in chromosome 10 of *D. rerio*. ^✠^Gene found in chromosome 6 of *D. rerio*.

**Figure S17**. Syntenic map of the submetacentric chromosome 4 of *Solea senegalensis* with respect to *Lepisosteus oculatus.* The % of the genes of BACs found in each chromosome of *L. oculatus* is shown between parentheses. This % was calculated taking into account the total number of the genes (69 genes) for chromosome 4 of *S. senegalensis*.

Legend symbols: ^+^Genes with low values in BLASTp analysis with *L. oculatus.* ^✦^Gene found in chromosome LG4 of *L. oculatus*. ^✧^Gene found in chromosome LG23 of *L. oculatus*.

**Figure S18**. Summary of repeat types present in chromosomes 2 and 4 of *Solea senegalensis*.

**Figure S19.** Kimura distance-based copy divergence analysis of transposable elements of chromosomes 2 and 4 of *Solea senegalensis* compared with their syntenic regions in other fish species. Graphs represent genome coverage (y axis) for each type of TEs (DNA transposons, SINE, LINE, and LTR retrotransposons) in the different genomes analyzed, clustered according to Kimura distances to their corresponding consensus sequence (x axis, K-value from 0 to 50). (**a**), (**c**), (**e**) and (**g**) results from chromosome 2, and (**b**), (**d**), (**f**) and (**h**) from chromosome 4. Rows are: *Cynoglossus semilaevis* (**a**, **b**), *Scophthalmus maximus* (**c**, **d**), *Oryzias latipes* (**e**, **f**) and *Danio rerio* (**g**, **h**).
