# Supplementary material for: Cytogenomics Unveil Possible Transposable Elements Driving Rearrangements in Chromosomes 2 and 4 of Solea senegalensis
Source: Int J Mol Sci. 2021 Feb 5;22(4):1614. doi: 10.3390/ijms22041614 (PMC7915175; doi:10.3390/ijms22041614)
Supplement: Supplementary file 1 [file ijms-22-01614-s001.zip › Table S1.docx]

**Table S1.** Summary of repeat types present in BAC sequences located in the chromosome 2 of *Solea senegalensis*. Coverage measured as percentage of repeat elements per BAC length.

| **COVERAGE** | **Length** | **Total** | **Retroelements** | **DNA transposons** | **Satellites** | **Simple repeats** | | **Low complexity** |
| --- | --- | --- | --- | --- | --- | --- | --- | --- |
| **52G10** | 173,182 | 8.5 | 1.08 | 2.35 | 0 | 4.69 | 0.37 | |
| **6P22** | 274,326 | 8.72 | 1.06 | 4.34 | 0.03 | 2.22 | 0.23 | |
| **60P19** | 176,179 | 5.25 | 0.43 | 2.29 | 0.02 | 1.99 | 0.33 | |
| **46C5** | 190,621 | 5.52 | 0.93 | 1.52 | 0.03 | 2.09 | 0.85 | |
| **36I3** | 34,866 | 8.54 | 1.68 | 3.86 | 0 | 2.6 | 0.41 | |
| **4D15** | 75,166 | 9.22 | 1.16 | 2.29 | 1.34 | 3.43 | 0.83 | |
| **38N10** | 203,362 | 8.13 | 2.11 | 4.02 | 0 | 1.73 | 0.24 | |
| **21O23** | 54,341 | 3.9 | 0.41 | 1.71 | 0 | 1.83 | 0.06 | |
