# Supplementary material for: Cytogenomics Unveil Possible Transposable Elements Driving Rearrangements in Chromosomes 2 and 4 of Solea senegalensis
Source: Int J Mol Sci. 2021 Feb 5;22(4):1614. doi: 10.3390/ijms22041614 (PMC7915175; doi:10.3390/ijms22041614)
Supplement: Supplementary file 1 [file ijms-22-01614-s001.zip › Table S2.docx]

**Table S2.** Summary of repeat types present in BAC sequences located in the chromosome 2 of *Solea senegalensis*. NL/Mb = Number of loci per Mb of BAC sequenced.

| **NL/Mb** | **Length** | **Retroelements** | **DNA transposons** | **Satellites** | **Simple repeats** | **Low complexity** |
| --- | --- | --- | --- | --- | --- | --- |
| **52G10** | 173,182 | 121.260 | 213.648 | 0.000 | 1189.500 | 51.968 |
| **6P22** | 274,326 | 94.778 | 422.855 | 3.645 | 597.829 | 51.034 |
| **60P19** | 176,179 | 56.760 | 278.126 | 5.676 | 624.365 | 62.436 |
| **46C5** | 190,621 | 94.428 | 215.086 | 5.246 | 503.617 | 110.166 |
| **36I3** | 34,866 | 258.131 | 286.812 | 0.000 | 717.031 | 114.725 |
| **4D15** | 75,166 | 106.431 | 279.382 | 39.912 | 944.576 | 66.519 |
| **38N10** | 203,362 | 196.694 | 427.809 | 0.000 | 476.982 | 54.091 |
| **21O23** | 54,341 | 55.207 | 257.632 | 0.000 | 515.265 | 18.402 |
