# Supplementary material for: Cytogenomics Unveil Possible Transposable Elements Driving Rearrangements in Chromosomes 2 and 4 of Solea senegalensis
Source: Int J Mol Sci. 2021 Feb 5;22(4):1614. doi: 10.3390/ijms22041614 (PMC7915175; doi:10.3390/ijms22041614)
Supplement: Supplementary file 1 [file ijms-22-01614-s001.zip › Table S3.docx]

**Table S3.** Summary of repeat types present in BAC sequences located in the chromosome 4 of *Solea senegalensis*. Coverage measured as percentage of repeat elements per BAC length.

| **COVERAGE** | **Length** | **Total** | **Retroelements** | **DNA transposons** | **Satellites** | **Simple repeats** | **Low complexity** |
| --- | --- | --- | --- | --- | --- | --- | --- |
| **12N15** | 162,898 | 5 | 1.82 | 1.53 | 0.05 | 1.53 | 0.14 |
| **3C15** | 79,261 | 4.95 | 0.82 | 1.92 | 0 | 1.66 | 0.15 |
| **46B2** | 177,377 | 5.56 | 1.17 | 2.39 | 0 | 1.58 | 0.15 |
| **30J4** | 372,766 | 5.84 | 1,42 | 2.11 | 0.02 | 2 | 0.23 |
| **12D24** | 183,576 | 6.06 | 1.05 | 2.72 | 0 | 1.84 | 0.27 |
| **8A23** | 100,967 | 5.18 | 1.11 | 2.15 | 0 | 1.55 | 0.37 |
| **46P22** | 93,963 | 24.39 | 10.37 | 6.52 | 0.04 | 7.37 | 0.55 |
| **36J2** | 56,196 | 11.1 | 1.78 | 4.15 | 0 | 4.72 | 0.67 |
| **36H2, 36H3** | 134,519 | 16.98 | 4.66 | 7.42 | 0 | 4.85 | 1.08 |
