# Supplementary material for: Cytogenomics Unveil Possible Transposable Elements Driving Rearrangements in Chromosomes 2 and 4 of Solea senegalensis
Source: Int J Mol Sci. 2021 Feb 5;22(4):1614. doi: 10.3390/ijms22041614 (PMC7915175; doi:10.3390/ijms22041614)
Supplement: Supplementary file 1 [file ijms-22-01614-s001.zip › Table S4.docx]

**Table S4.** Summary of repeat types present in BAC sequences located in the chromosome 4 of *Solea senegalensis*. NL/Mb = Number of loci per Mb of BAC sequenced.

| **NL/Mb** | **Length** | **Retroelements** | **DNA transposons** | **Satellites** | **Simple repeats** | **Low complexity** |
| --- | --- | --- | --- | --- | --- | --- |
| **12N15** | 162,898 | 79.805 | 165.748 | 12.278 | 417.439 | 30.694 |
| **3C15** | 79,261 | 88.316 | 264.947 | 0.000 | 492.045 | 37.850 |
| **46B2** | 177,377 | 112.754 | 202.958 | 0.000 | 422.828 | 39.464 |
| **30J4** | 372,766 | 99.258 | 252.169 | 2.683 | 560.673 | 32.192 |
| **12D24** | 183,576 | 92.605 | 256.025 | 0.000 | 522.944 | 59.921 |
| **8A23** | 100,967 | 89.138 | 297.127 | 0.000 | 455.594 | 89.138 |
| **46P22** | 93,963 | 340.560 | 776.902 | 10.642 | 1628.301 | 95.782 |
| **36J2** | 56,196 | 195.743 | 373.692 | 0.000 | 1138.871 | 124.564 |
| **36H2, 36H3** | 134,519 | 327.091 | 475.769 | 0.000 | 899.501 | 118.942 |
