# Supplementary figures and images for: Cytogenomics Unveil Possible Transposable Elements Driving Rearrangements in Chromosomes 2 and 4 of Solea senegalensis
Source: Int J Mol Sci. 2021 Feb 5;22(4):1614. doi: 10.3390/ijms22041614 (PMC7915175; doi:10.3390/ijms22041614)

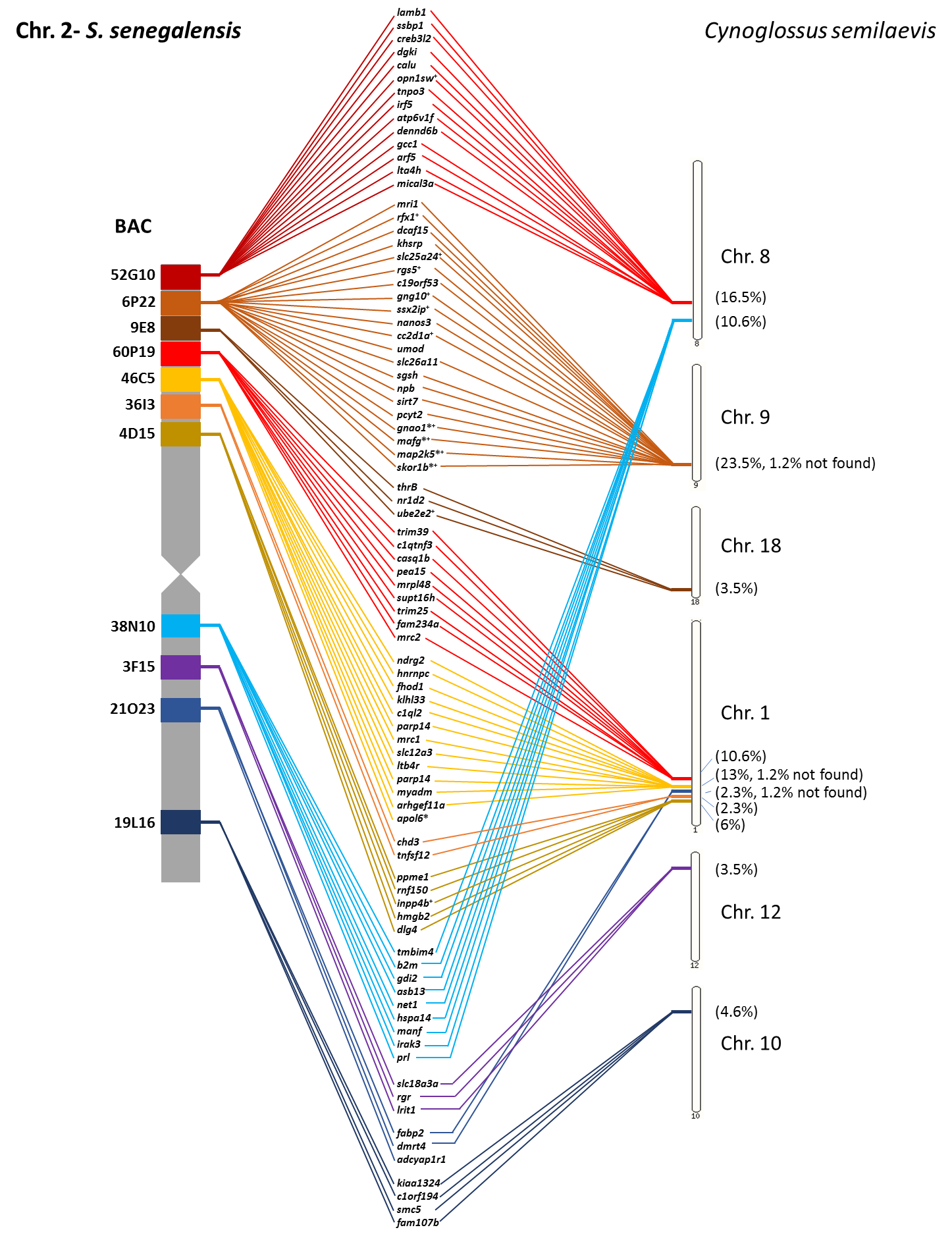

Supplement: Supplementary file 1 [file ijms-22-01614-s001.zip › Figure S1.png]

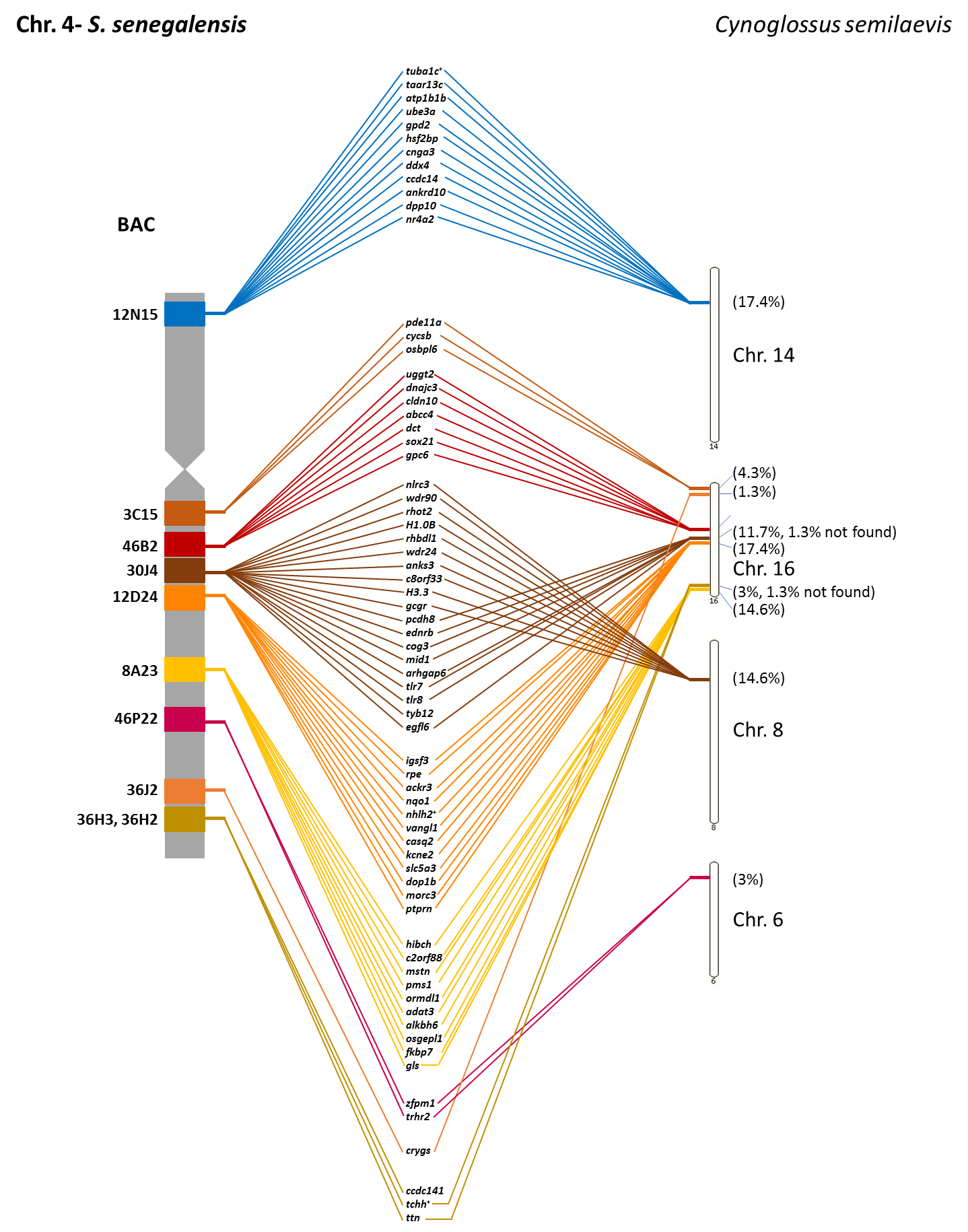

Supplement: Supplementary file 1 [file ijms-22-01614-s001.zip › Figure S10.png]

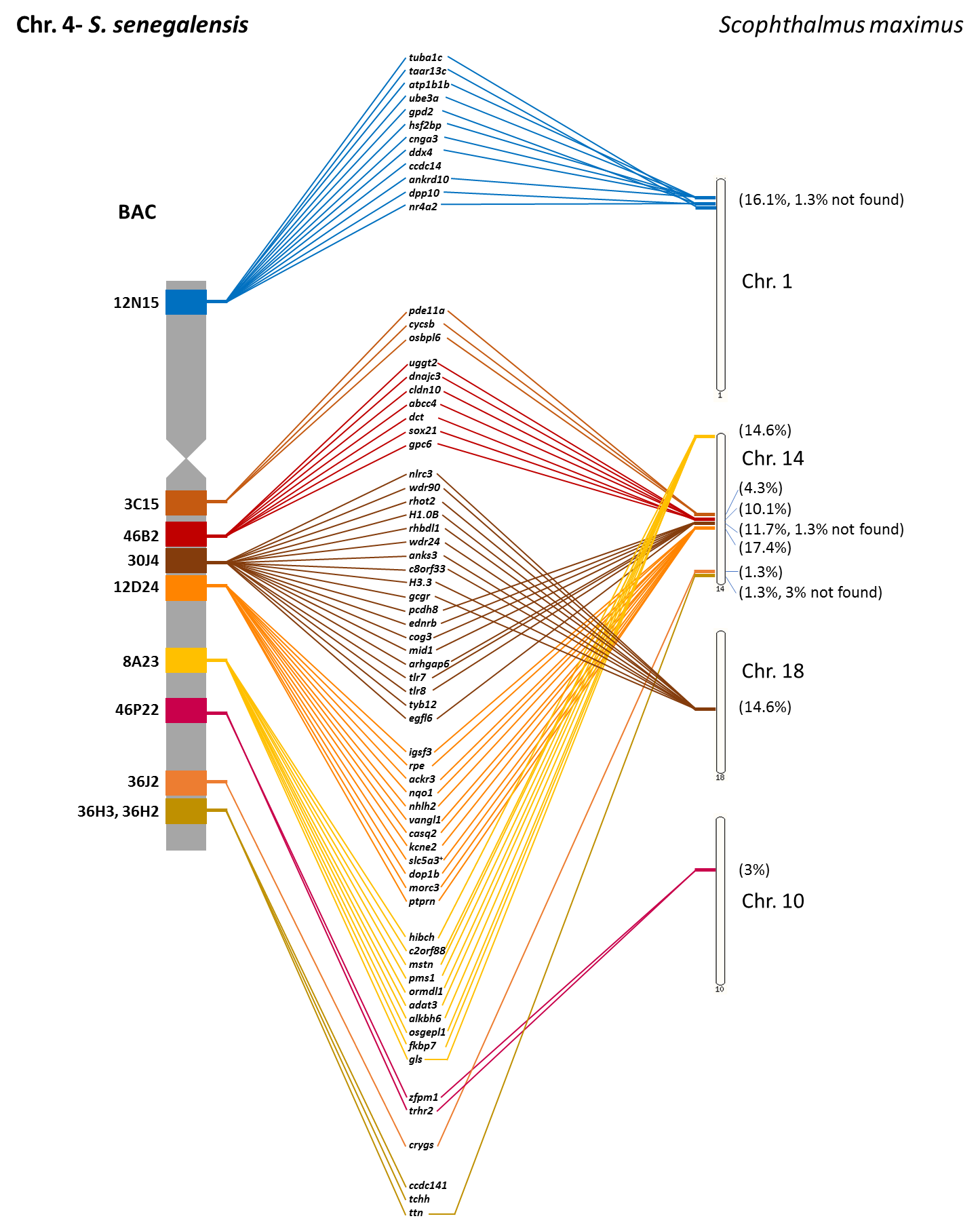

Supplement: Supplementary file 1 [file ijms-22-01614-s001.zip › Figure S11.png]

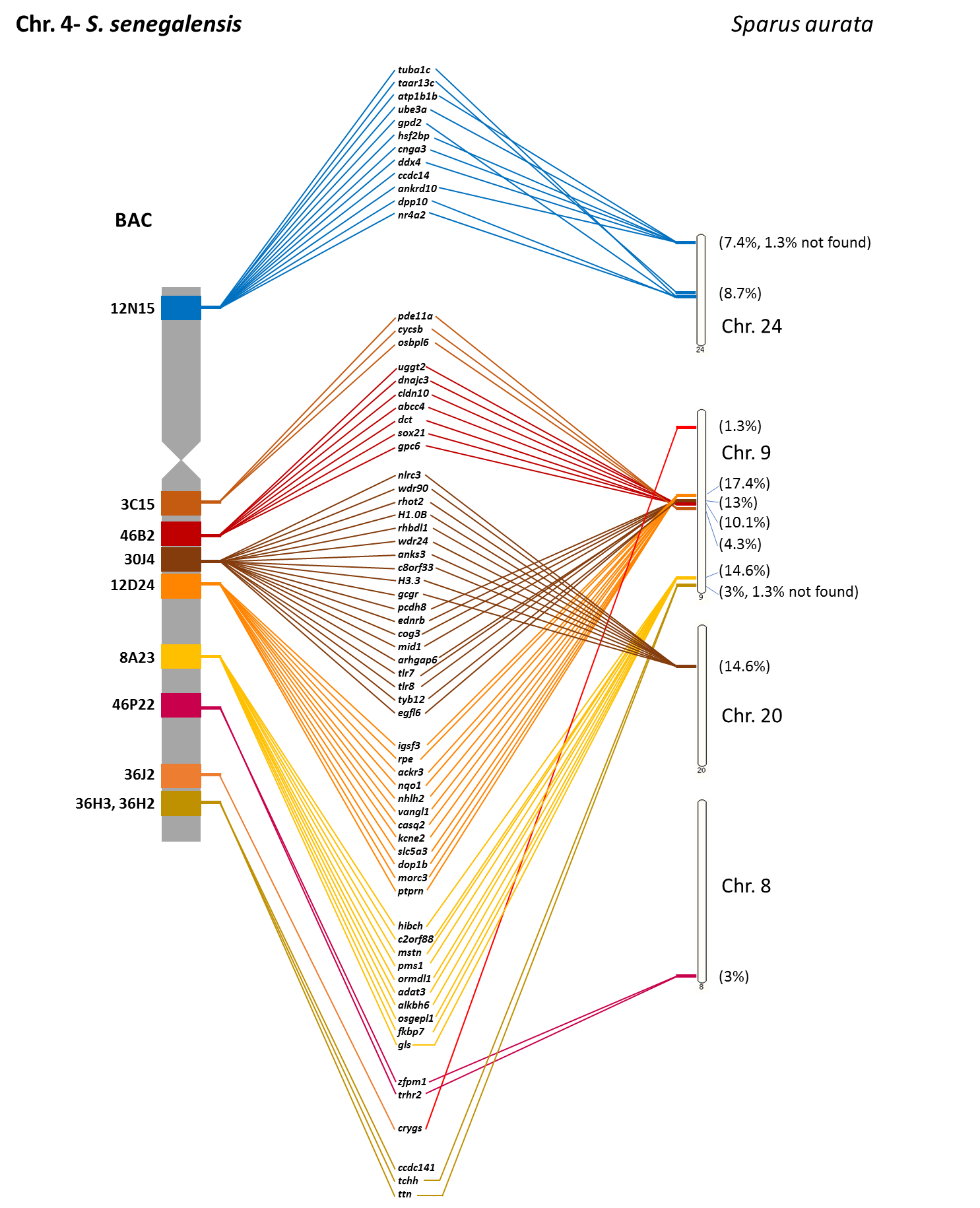

Supplement: Supplementary file 1 [file ijms-22-01614-s001.zip › Figure S12.png]

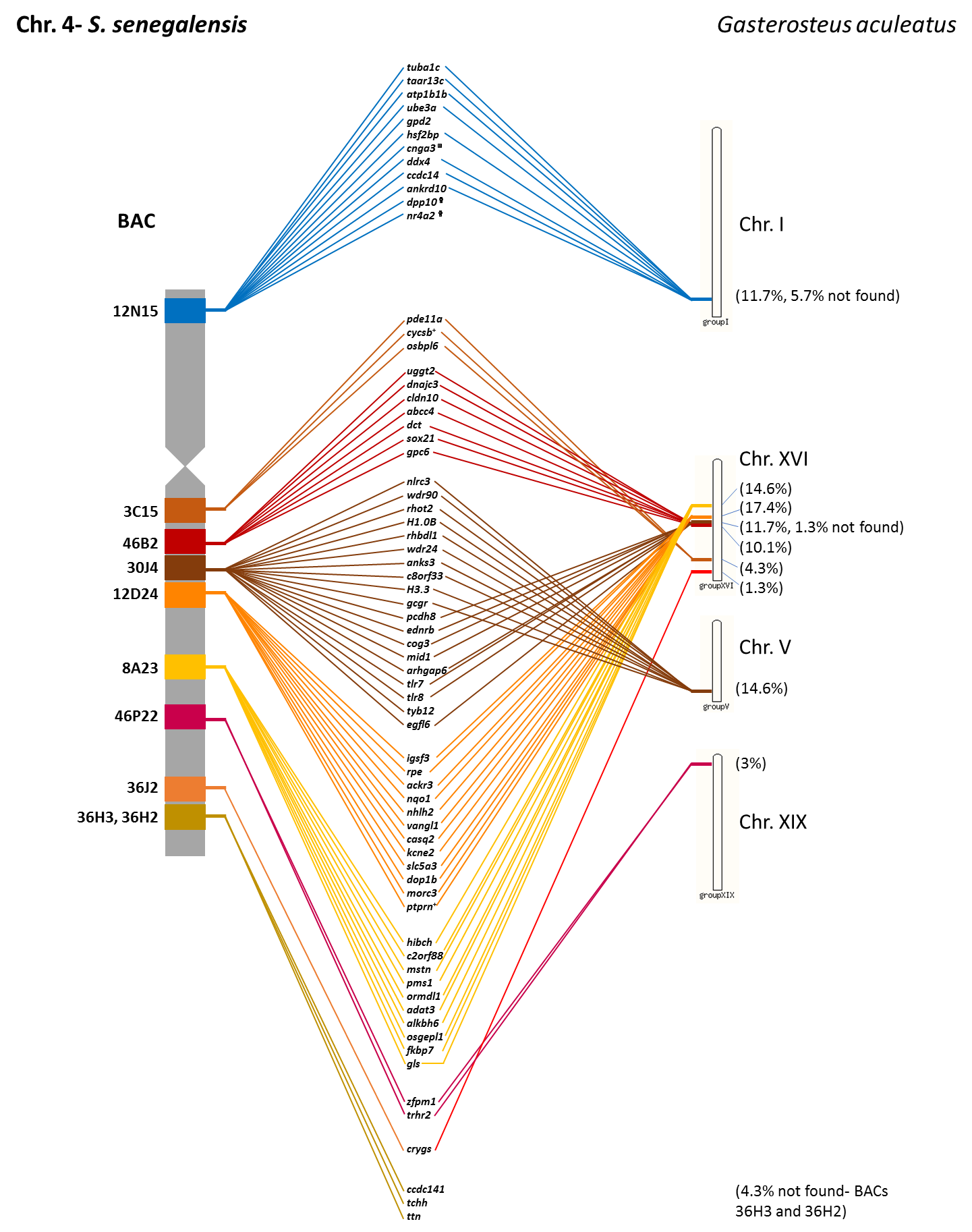

Supplement: Supplementary file 1 [file ijms-22-01614-s001.zip › Figure S13.png]

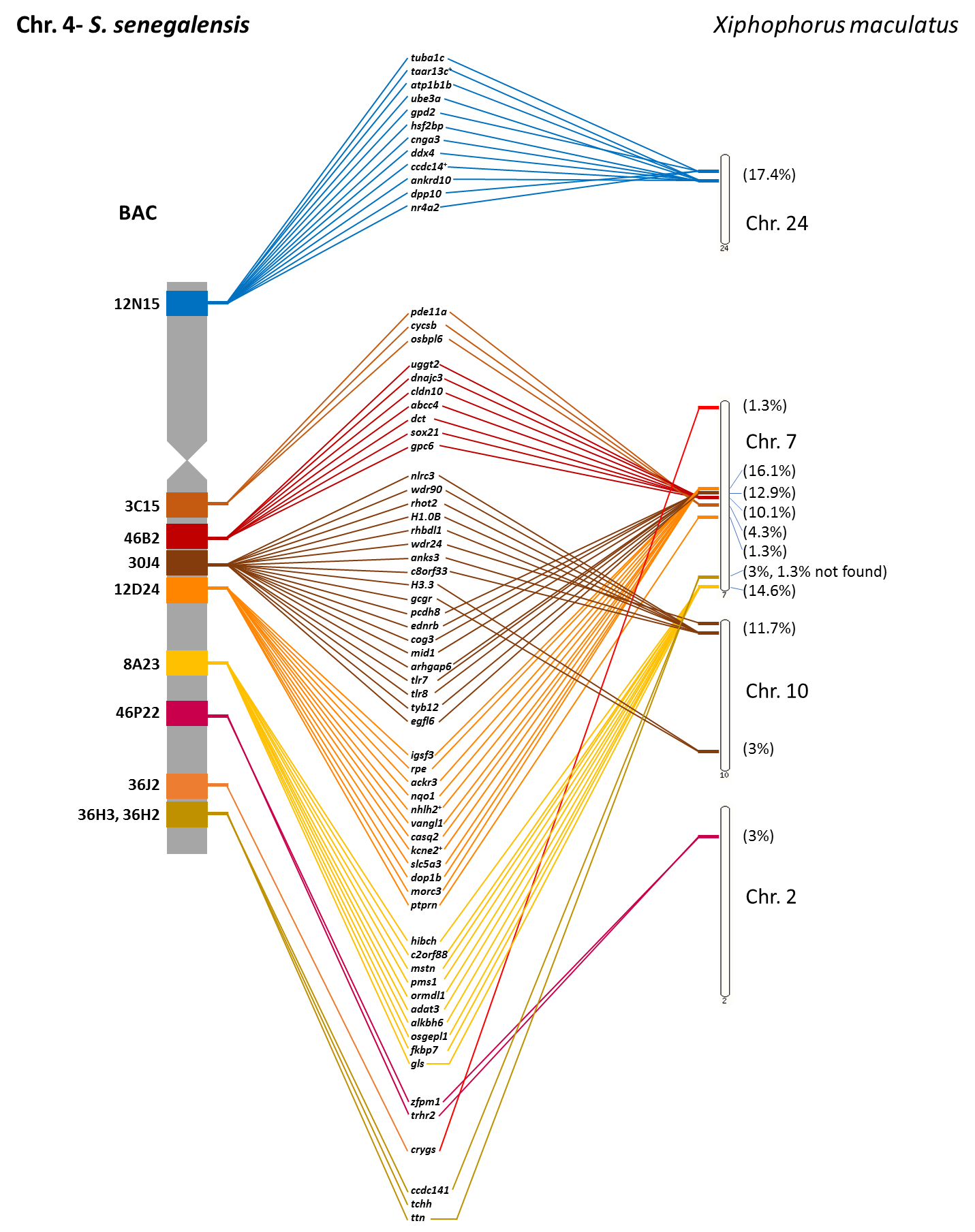

Supplement: Supplementary file 1 [file ijms-22-01614-s001.zip › Figure S14.png]

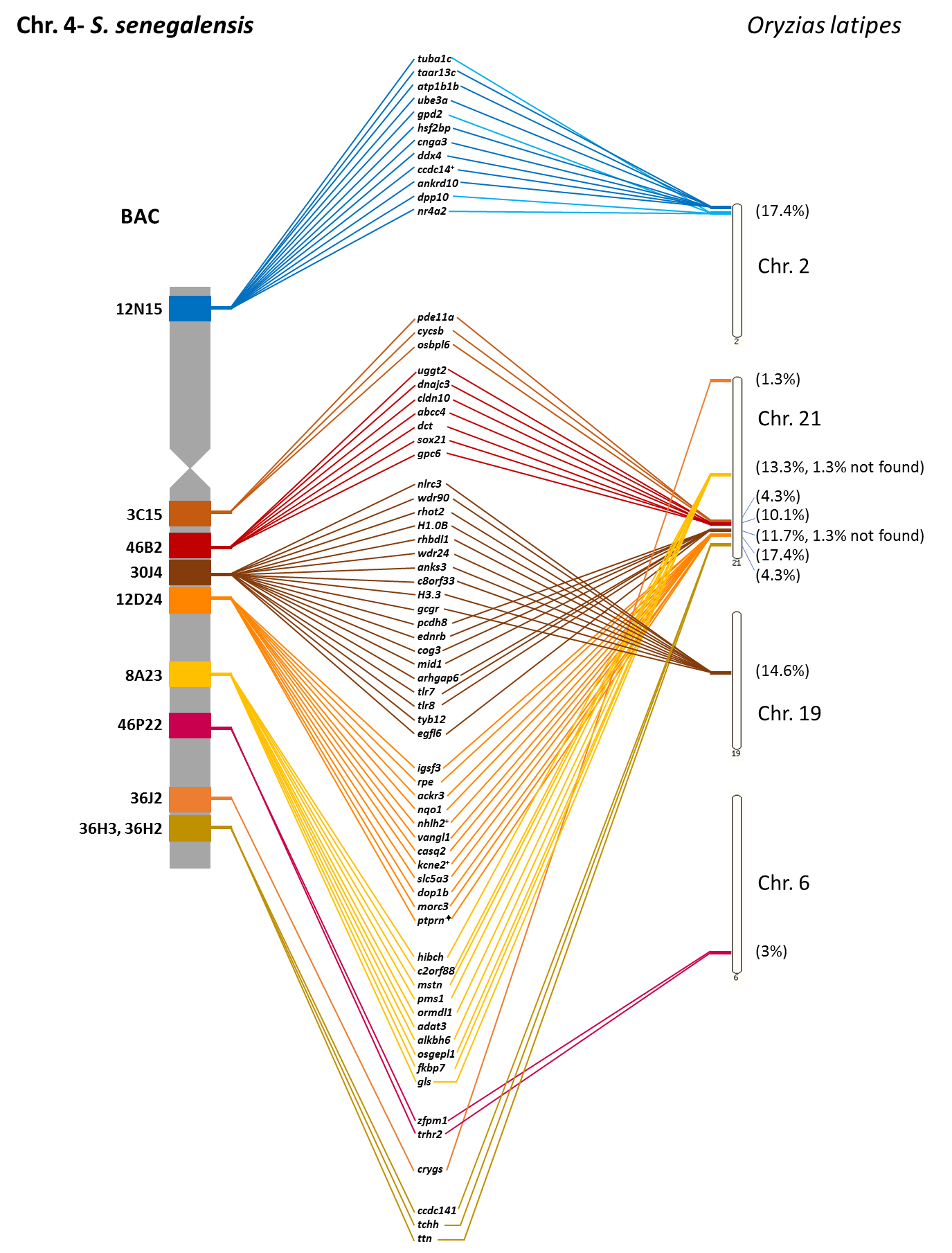

Supplement: Supplementary file 1 [file ijms-22-01614-s001.zip › Figure S15.png]

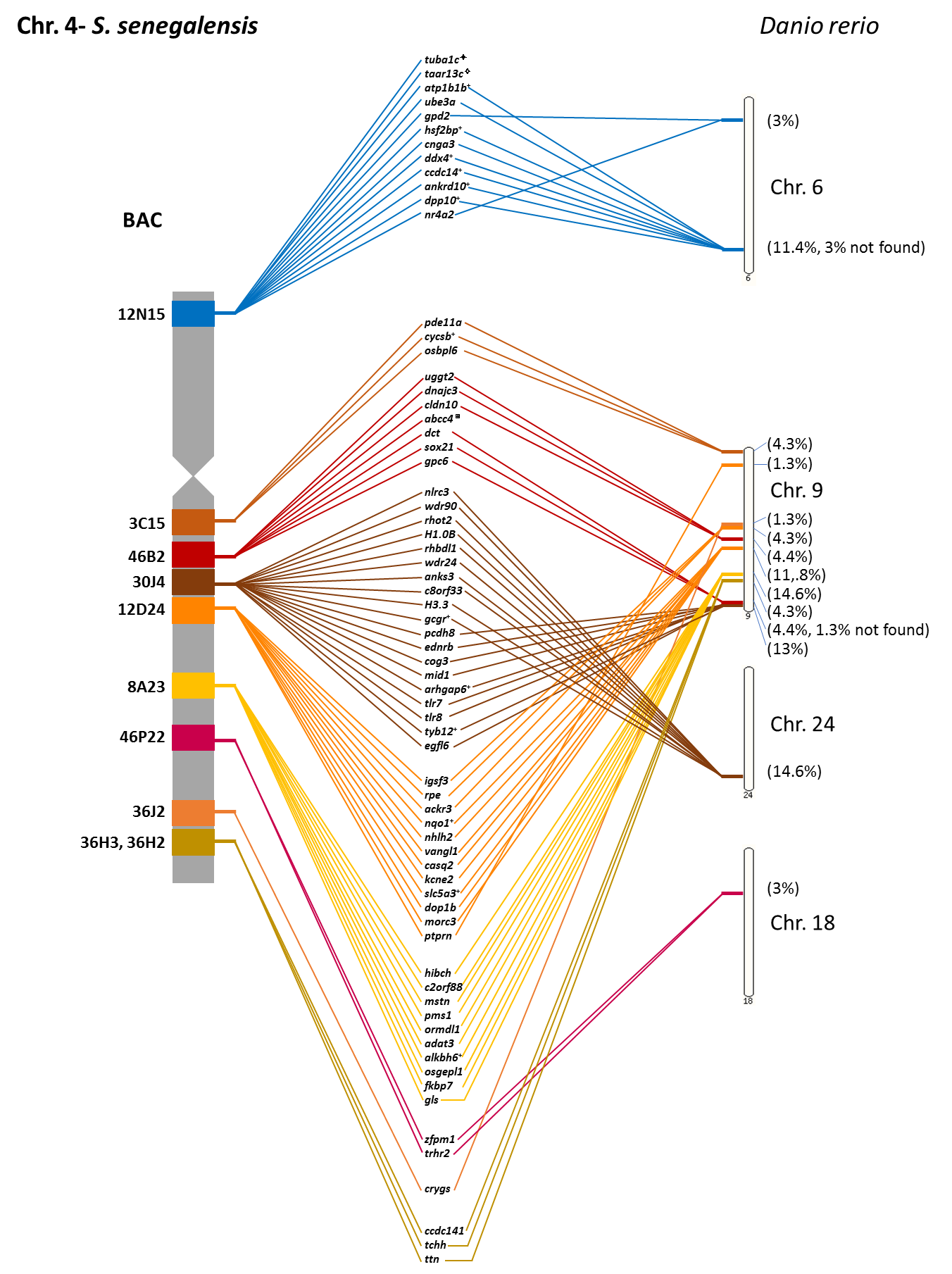

Supplement: Supplementary file 1 [file ijms-22-01614-s001.zip › Figure S16.png]

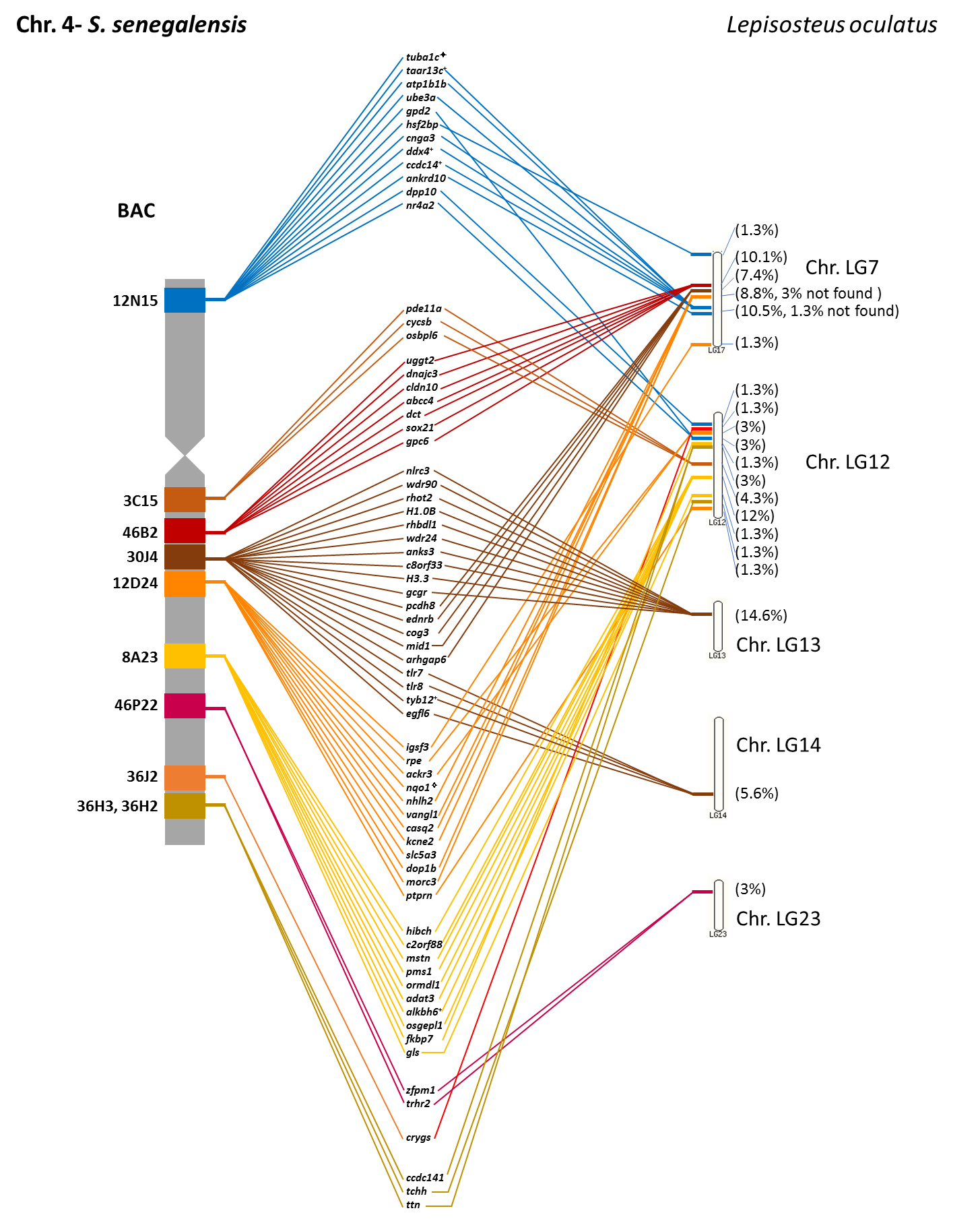

Supplement: Supplementary file 1 [file ijms-22-01614-s001.zip › Figure S17.png]

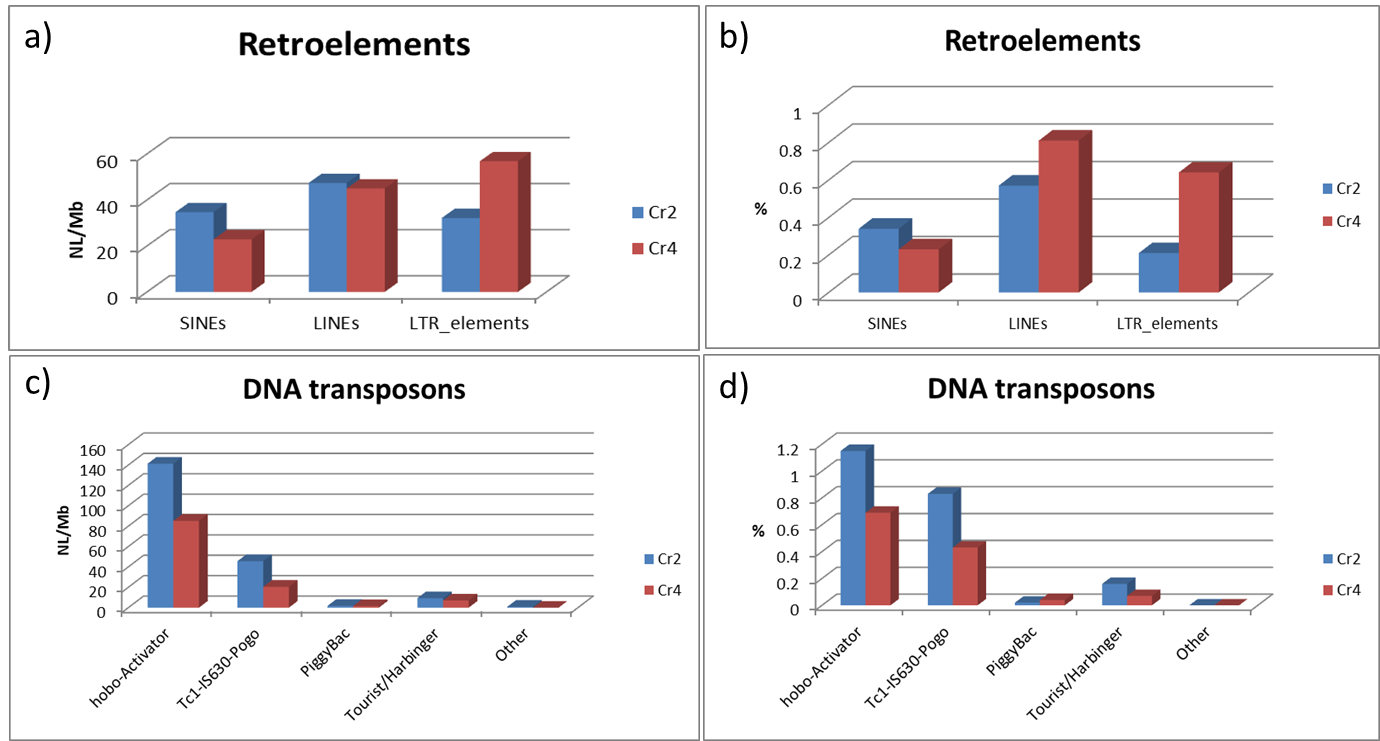

Supplement: Supplementary file 1 [file ijms-22-01614-s001.zip › Figure S18.png]

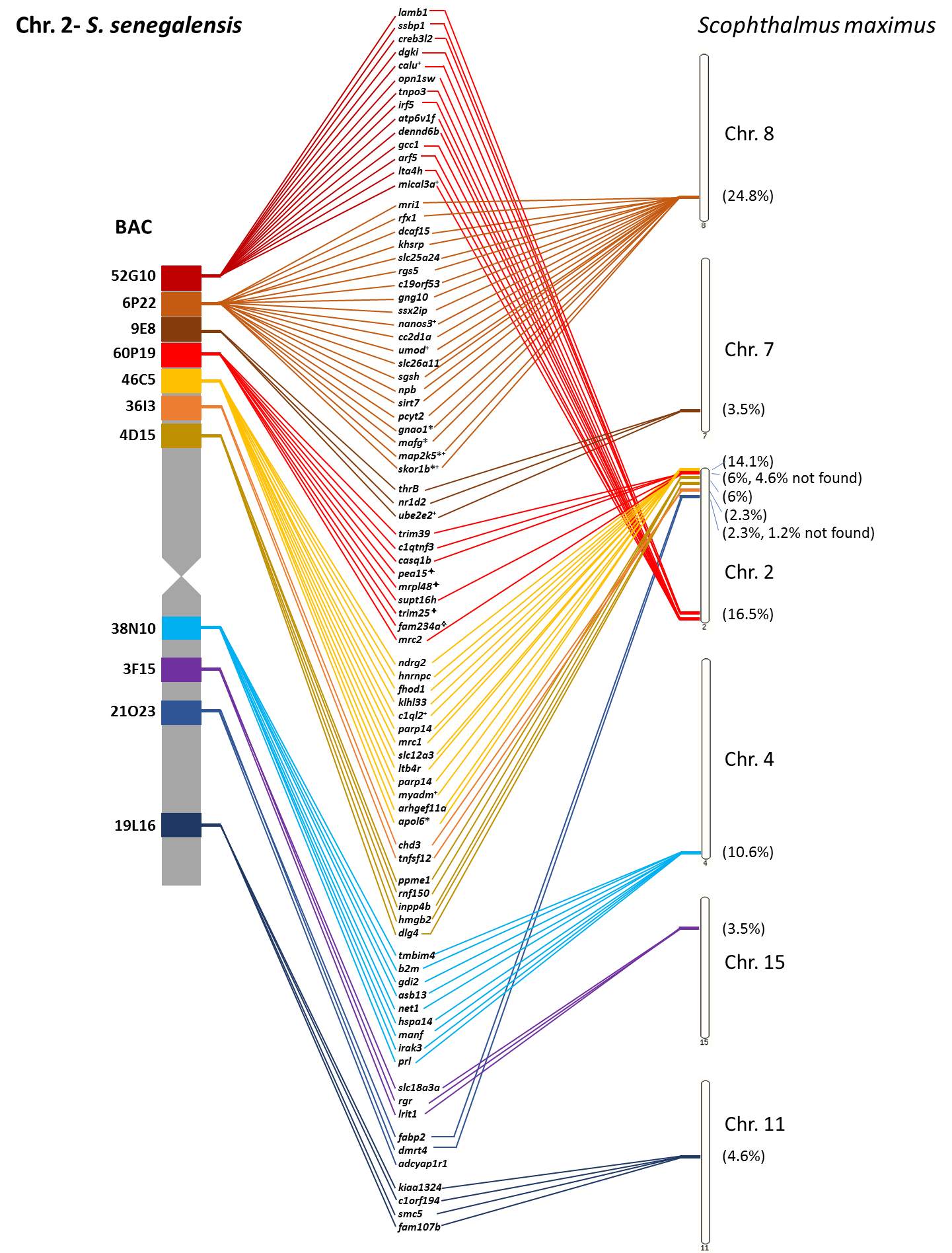

Supplement: Supplementary file 1 [file ijms-22-01614-s001.zip › Figure S2.png]

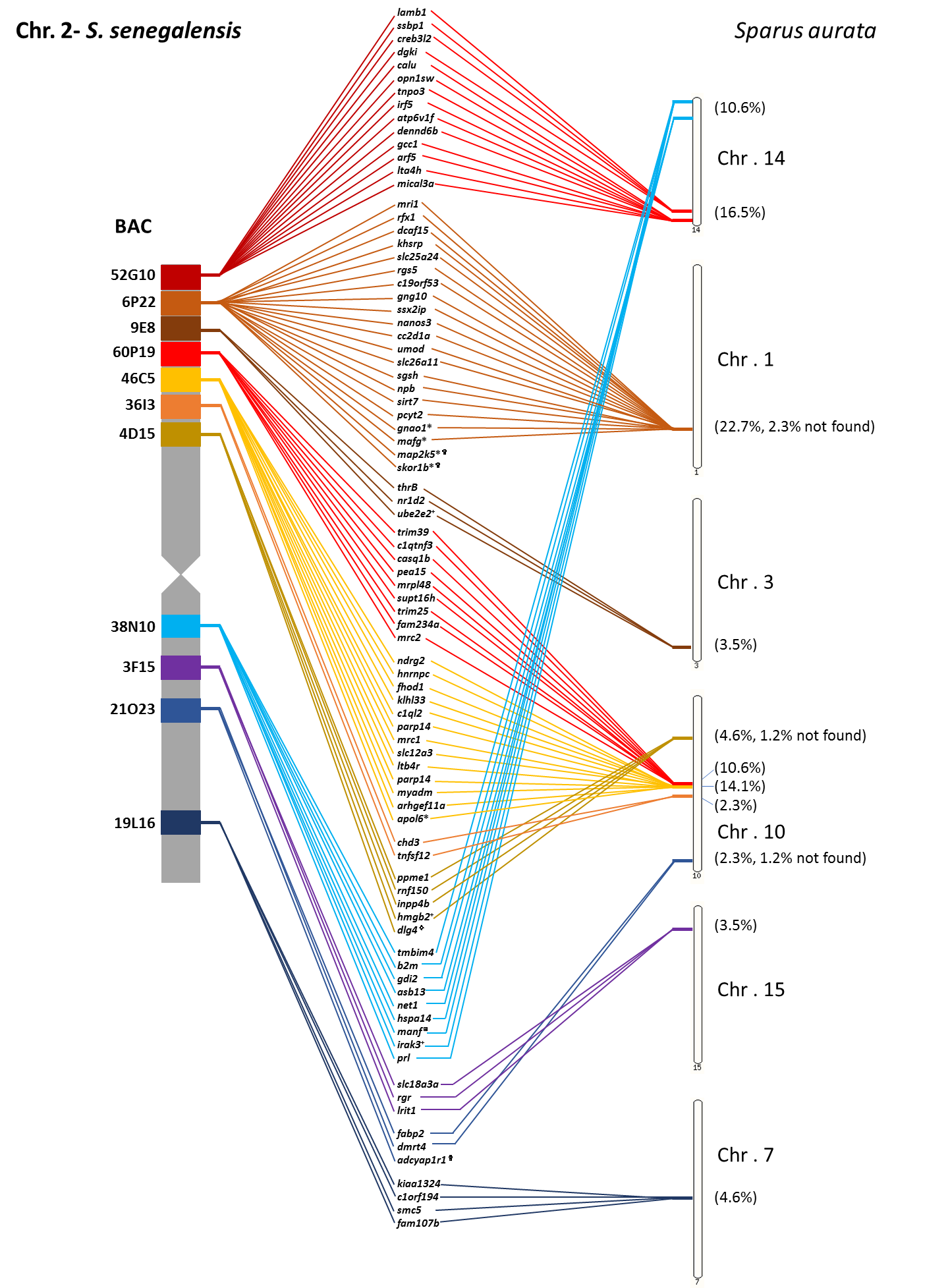

Supplement: Supplementary file 1 [file ijms-22-01614-s001.zip › Figure S3.png]

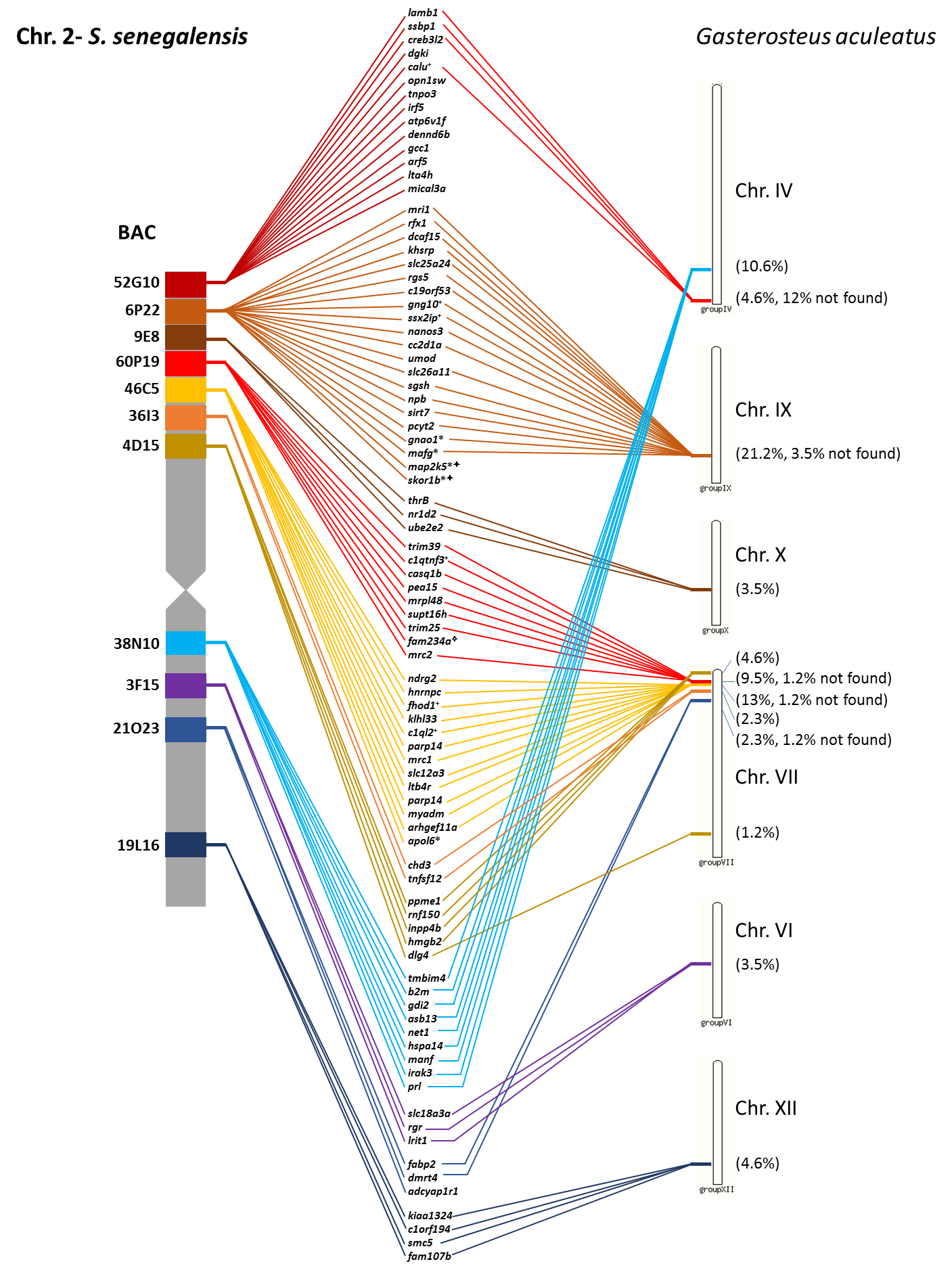

Supplement: Supplementary file 1 [file ijms-22-01614-s001.zip › Figure S4.png]

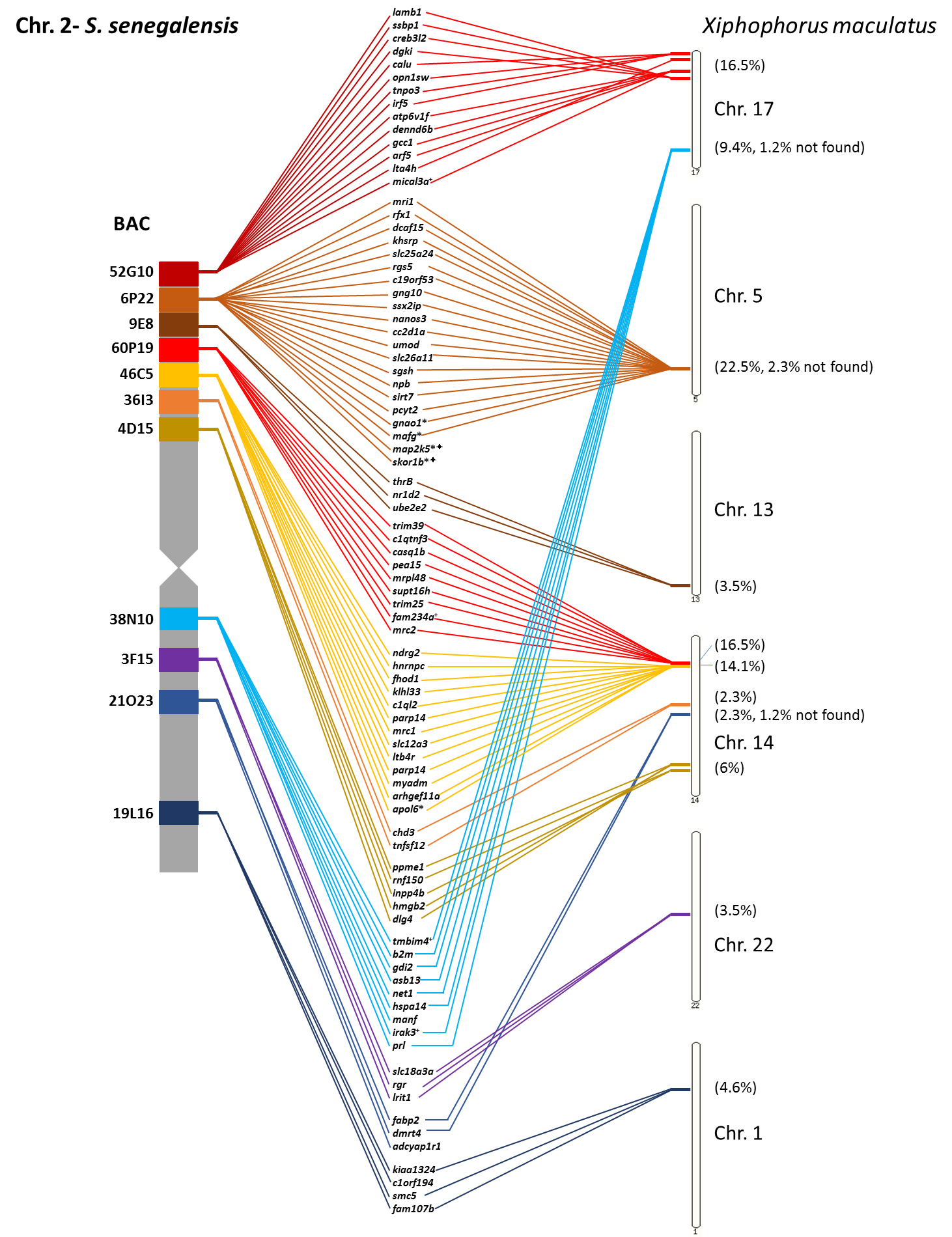

Supplement: Supplementary file 1 [file ijms-22-01614-s001.zip › Figure S5.png]

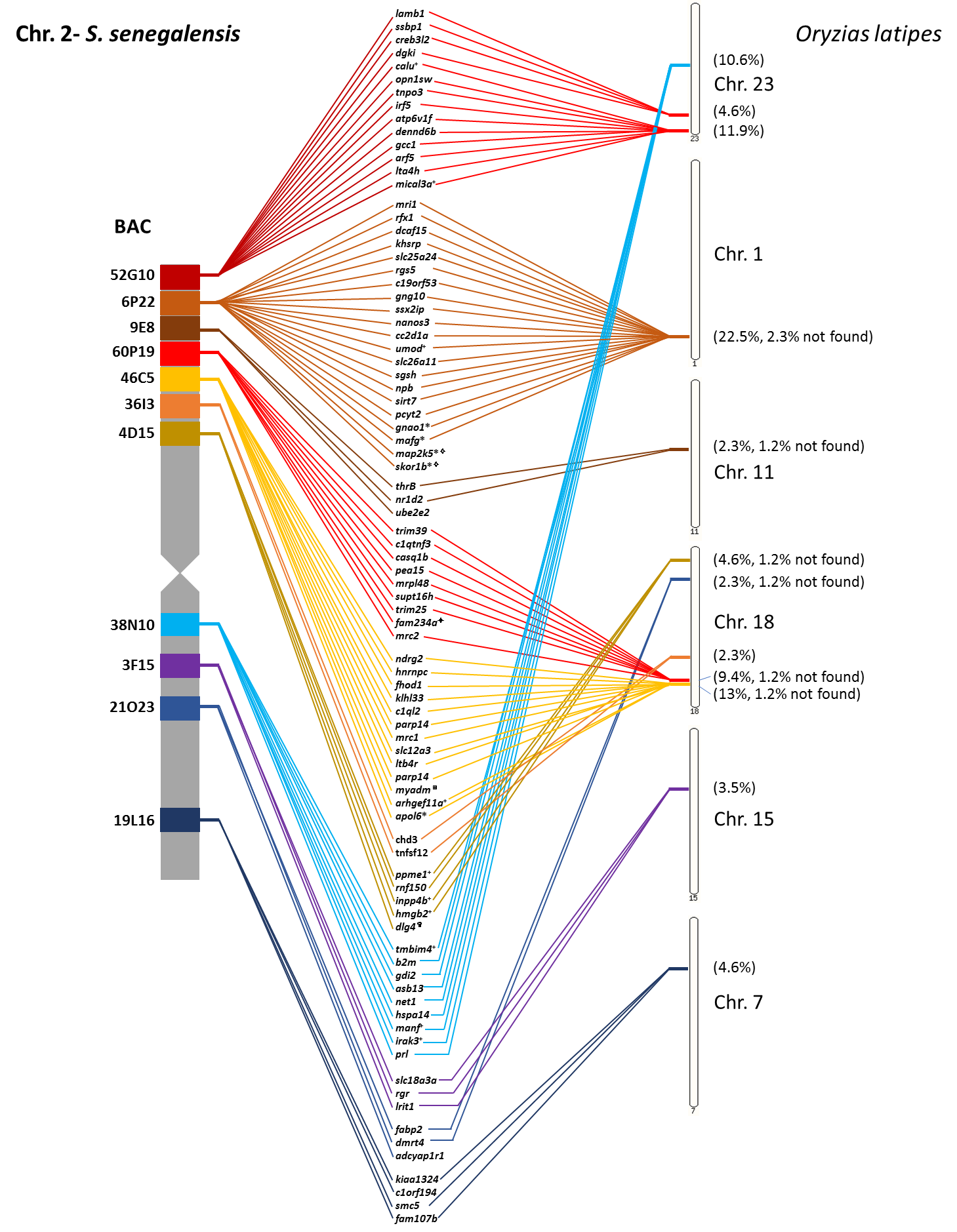

Supplement: Supplementary file 1 [file ijms-22-01614-s001.zip › Figure S6.png]

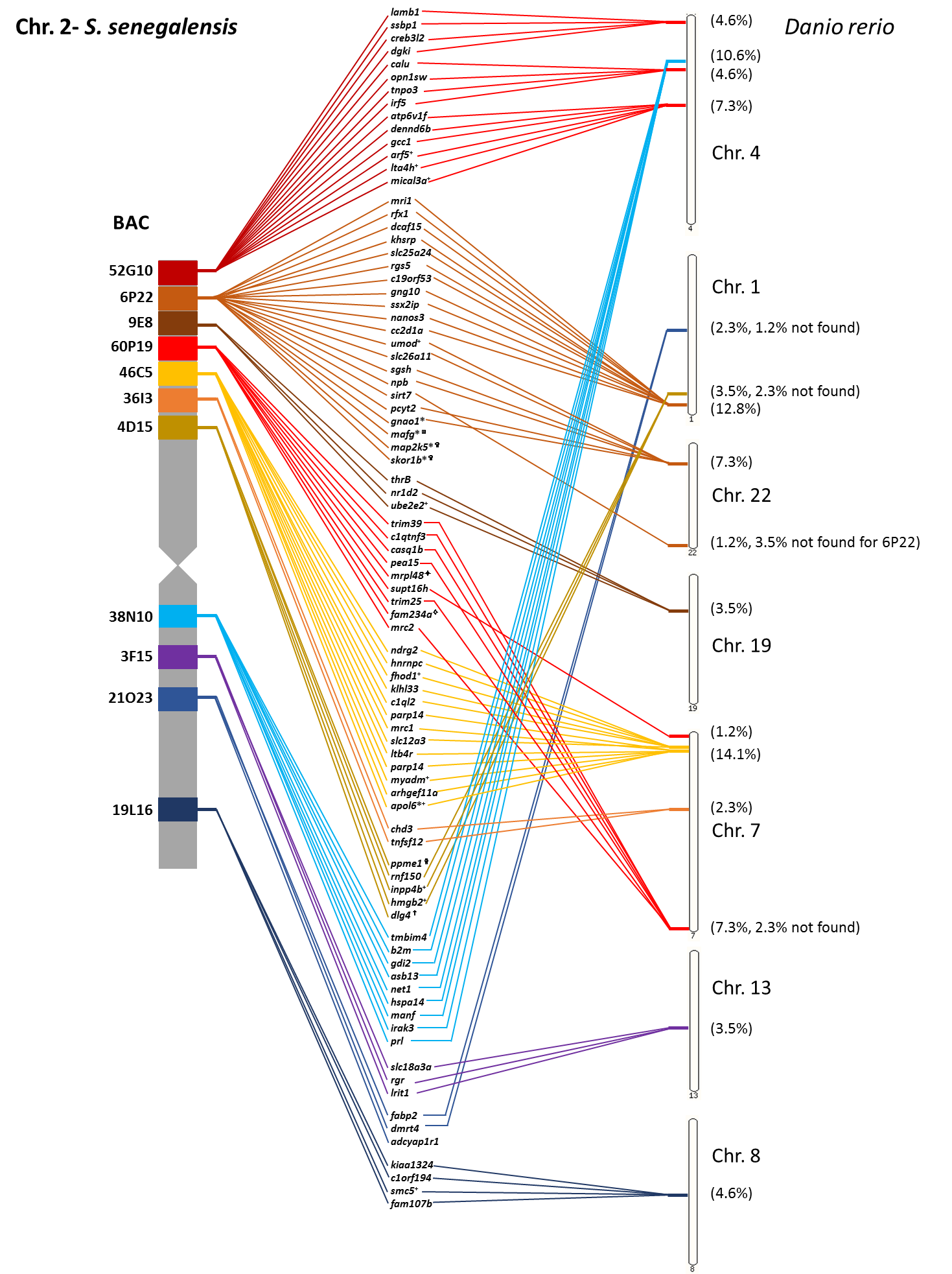

Supplement: Supplementary file 1 [file ijms-22-01614-s001.zip › Figure S7.png]

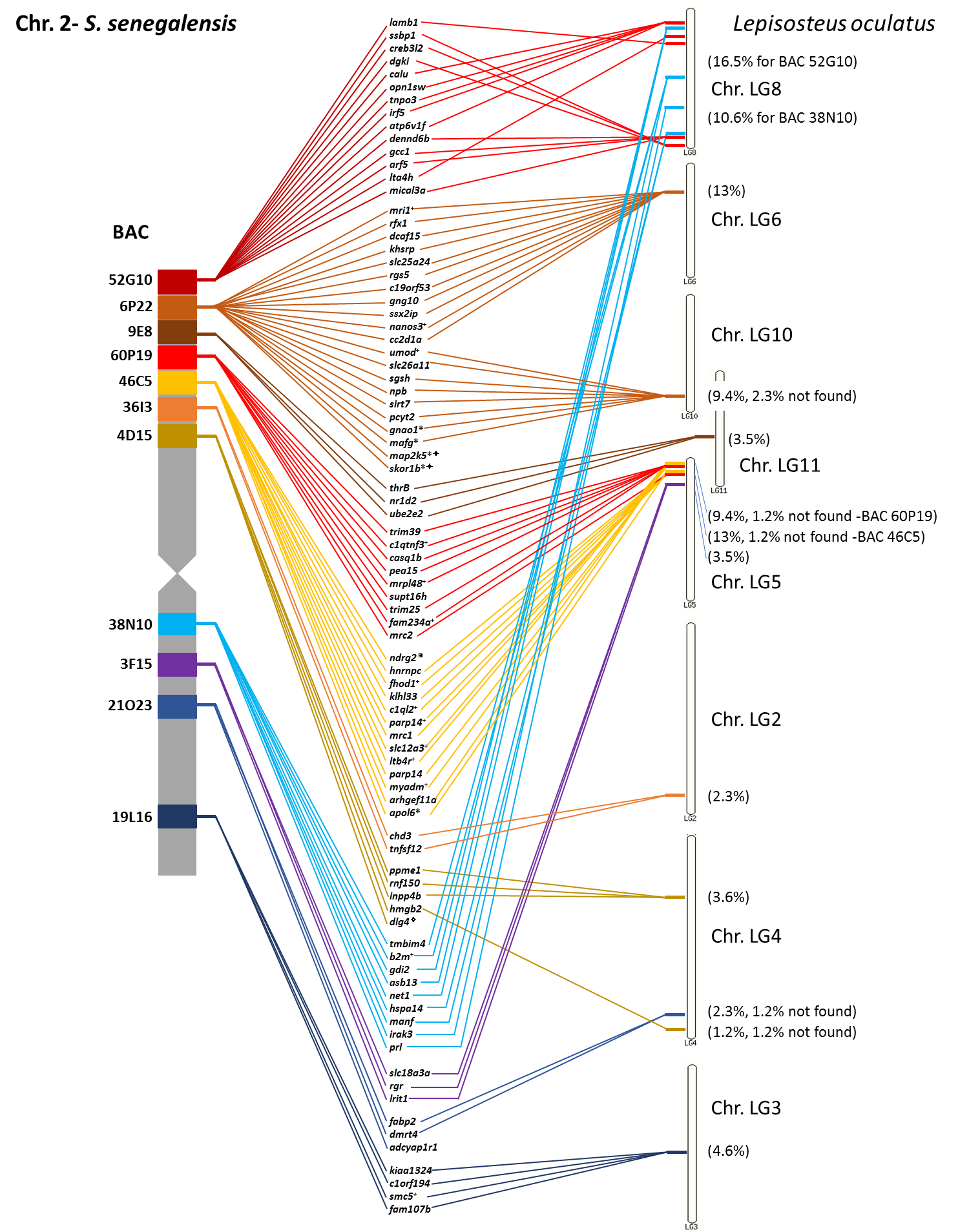

Supplement: Supplementary file 1 [file ijms-22-01614-s001.zip › Figure S8.png]
